# Supplementary material for: Effectiveness of a behavioural intervention involving regular weighing and feedback by community midwives within routine antenatal care to prevent excessive gestational weight gain: POPS2 randomised controlled trial
Source: BMJ Open. 2019 Sep 17;9(9):e030174. doi: 10.1136/bmjopen-2019-030174 (PMC6756421; doi:10.1136/bmjopen-2019-030174)
Supplement: Supplementary data [file bmjopen-2019-030174supp001.pdf]

Supplementary Table 1: Maternal and neonatal complications by group

	Intervention	Usual care
**Mother:**	N (%)	N (%)
Caesarean section	68/304 (22.4)	69/302 (22.8)
Length of inpatient stay mean (sd)<br>median (IQR)	1.7 (1.5) 305<br>1.0 (1.0 to 2.0)	1.7 (1.7) 306<br>1.0 (1.0 to 2.0)
Maternal ICU admission	n/a	n/a
Preeclampsia	6/315 (1.9)	8/317 (2.5)
Pregnancy induced hypertension	n/a	n/a
Gestational diabetes	12/315 (3.8)	17/317 (5.4)
Maternal sepsis	n/a	n/a
Preterm delivery	17/304 (5.6)	14/302 (4.6)
Miscarriage	5/317 (1.6)	1/316 (0.3)
Stillbirth	0/316 (0)	1/316 (0.3)
Shoulder dystocia	3/312 (1.0)	2/314 (0.6)
**Baby:**		
Treatment for jaundice	34/312 (10.9)	27/314 (8.6)
Low Apgar score (<7) at 1 min<br>Low apgar score (<7) at 5 mins	n/a<br>4/254 (1.6)	n/a<br>1/249 (0.4)
Admission to NICU	26/261 (10.0)	21/262 (8.0)
Neonatal death	n/a	n/a
Neonatal sepsis	n/a	n/a
Gestational age (wks) mean (sd)<br>median (IQR)	39.2 (2.1) 304<br>40 (39-40)	39.3 (1.6) 302<br>40 (38-40)
Birth weight (g) mean (sd)<br>median (IQR)	3348.6 (567.1) 304<br>3373.5 (3060-3665)	3401.8 (550.7) 301<br>3460 (3040-3745)

Supplementary Table 2: Per-protocol analysis

	**Intervention**	**Usual care**	**Intervention-Usual care**		
	**Number exceeding IOM guideline/N$(%)**	**Number exceeding IOM guideline/N$(%)**	**% (95% CI)***	**Adjusted odds ratio (95% CI)***	**P value**
Women recorded weight weekly >70% of time during pregnancy	7/46 (15.2)	67/254 (26.4)	-9.2<br>(-26.3, 8.0)	0.58<br>(0.23, 1.47)	0.25
Midwife overall accuracy for >70% appointments	8/42 (19.0)	67/254 (26.4)	-5.9<br>(-28.0, 16.2)	0.71<br>(0.27, 1.88)	0.49
Women weighing themselves 5 or more times during pregnancy	18/95 (18.9)	67/254 (26.4)	-7.0<br>(-23.2, 9.2)	0.68<br>(0.35, 1.33)	0.26

$ Includes objective and self-reported weights
*adjusted by Site, BMI category and midwife (random effect)
