# Supplementary material for: Effectiveness of a behavioural intervention involving regular weighing and feedback by community midwives within routine antenatal care to prevent excessive gestational weight gain: POPS2 randomised controlled trial
Source: BMJ Open. 2019 Sep 17;9(9):e030174. doi: 10.1136/bmjopen-2019-030174 (PMC6756421; doi:10.1136/bmjopen-2019-030174)
Supplement: Author's manuscript [file bmjopen-2019-030174.draft_revisions.pdf]

BMJ Open is committed to open peer review. As part of this commitment we make the peer review history of every article we publish publicly available.

When an article is published we post the peer reviewers' comments and the authors' responses online. We also post the versions of the paper that were used during peer review. These are the versions that the peer review comments apply to.

The versions of the paper that follow are the versions that were submitted during the peer review process. They are not the versions of record or the final published versions. They should not be cited or distributed as the published version of this manuscript.

BMJ Open is an open access journal and the full, final, typeset and author-corrected version of record of the manuscript is available on our site with no access controls, subscription charges or pay-per-view fees (http://bmjopen.bmj.com).

If you have any questions on BMJ Open's open peer review process please email info.bmjopen@bmj.com

BMJ Open

# Effectiveness of a behavioural intervention involving regular weighing and feedback by community midwives within routine antenatal care to prevent excessive gestational weight gain: POPS2 randomised controlled trial

| | |
|---|---|
| Journal: | *BMJ Open* |
| Manuscript ID | bmjopen-2019-030174 |
| Article Type: | Research |
| Date Submitted by the Author: | 08-Mar-2019 |
| Complete List of Authors: | Daley, Amanda; Loughborough University, School of Sport, Exercise and Health Sciences<br>Jolly, Kate; University of Birmingham<br>Jebb, Susan; Univerof Oxford, Primary Care Health Sciences<br>Roalfe, Andrea; Oxford University<br>Mackilllop, Lucy; John Radcliffe Hospital<br>Lewis, Amanda; University of Bristol<br>Clifford, Sue; University of Birmingham College of Medical and Dental Sciences<br>Usman, Muhammad; University Hospitals of Leicester NHS Trust<br>Ohadike, Corah; Oxford University<br>Kenyon, Sara; University of Birmingham, Public Health, Epidemiology and Biostatistics<br>MacArthur, Christine; University of Birmingham, Public Health<br>Aveyard, Paul; University of Oxford, Primary Care Health Sciences |
| Keywords: | pregnancy, weight, weighing |
| | |

SCHOLARONE™
Manuscripts

# Effectiveness of a behavioural intervention involving regular weighing and feedback by community midwives within routine antenatal care to prevent excessive gestational weight gain: POPS2 randomised controlled trial

**Short Title: Routine weighing during pregnancy by community midwives**


Amanda J Daley[1] PhD, a.daley@lboro.ac.uk
Kate Jolly[2] PhD, c.b.jolly@bham.ac.uk
Susan A Jebb[3]PhD, susan.jebb@phc.ox.ac.uk
Andrea K Roalfe[3] MSc, andrea.roalfe@phc.ox.ac.uk
Lucy Mackillop[4] BM BCh, lucy.mackillop@obs-gyn.ox.ac.uk
Amanda L Lewis[5] PhD, amanda.lewis@bristol.ac.uk
Sue J Clifford[2] BSc, s.clifford@bham.ac.uk
Muhammad Usman[2] MSc, musman.bstat@gmail.com
Corah O Ohadike[4] BMBS, corah.ohadike@wrh.ox.ac.uk
Sara Kenyon[2] PhD, s.kenyon@bham.ac.uk
Christine MacArthur[2] PhD, c.macarthur@bham.ac.uk
Paul Aveyard[3] PhD, paul.aveyard@phc.ox.ac.uk

[1]School of Sport, Exercise and Health Sciences, University of Loughborough, Epinal Way, Loughborough, Leicestershire, LE11 3TU

[2]Institute for Applied Health Research, College of Medical and Dental Sciences, University of Birmingham, Birmingham, B15 2TT

[3]Nuffield Department of Primary Care Health Sciences, University of Oxford, Radcliffe Observatory Quarter, Woodstock Road, Oxford, OX2 6GG

[4]Nuffield Department of Obstetrics and Gynaecology, University of Oxford, John Radcliffe Hospital, Headley Way, Oxford, OX3 9DU

[5]School of Social and Community Medicine, University of Bristol, Canynge Hall, 39 Whatley Road, Bristol, BS8 2PS

**Corresponding author**
Professor Amanda Daley
School of Sport, Exercise and Health Sciences
University of Loughborough
Epinal Way
Loughborough
Leicestershire, LE11 3TU
a.daley@lboro.ac.uk


Word count: 3,700 words

For peer review only

**Abstract**

**Objectives:** To assess the effectiveness of a brief behavioural intervention based on routine antenatal weighing to prevent excessive gestational weight gain (defined by United States Institute of Medicine).

**Design:** Randomised controlled trial

**Setting:** Antenatal clinical in England

**Participants:** Women between $10^{+0}$ and $14^{+6}$ weeks gestation, not requiring specialist obstetric care,

**Interventions:** Participants were randomised to usual antenatal care or usual care plus the intervention. The intervention involved community midwives weighing women at antenatal appointments, setting maximum weight gain limits between appointments and providing brief feedback. Women were encouraged to monitor and record their own weight weekly to assess their progress against the maximum limits set by their midwife. The comparator was usual maternity care.

**Primary and secondary outcome measures:** Excessive gestation weight gain, depression and anxiety

**Results** 656 women from four maternity centres were recruited; 329 randomised to the intervention group and 327 to usual care. We found no evidence that the intervention decreased excessive gestational weight gain. At 38 weeks gestation the proportions gaining excessive gestational weight were 27.6% versus 28.9% (adjusted odds ratio 0.84, 95% CI: 0.53-1.33) in the intervention and usual care group respectively. There were no significant differences between the groups in anxiety and depression scores (anxiety: adjusted mean -0.50, 95% CI:-1.17-0.16; depression: adjusted mean -0.59, 95% CI:-1.23-0.05).

**Conclusions**: A behavioural intervention delivered by community midwives involving routine weighing throughout pregnancy, setting maximum weight gain targets, and encouraging women

For peer review only

to weigh themselves each week to check progress did not prevent excessive gestational weight gain. There was no evidence of psychological harm.

**Trial registration:** Current Controlled Trials registry number ISRCTN 67427351. 29th October 2014. http://www.isrctn.com/ISRCTN67427351.

**Key words**: pregnancy, weighing, midwives, weight

**Strength and limitations of this study**

Most (~76%) eligible women participated in the trial, meaning the results reflect the impact in the general population.

A relatively large proportion of women were recruited from non-White ethnic groups and/or low socio-economic backgrounds

Weight was objectively assessed and we trained over 100 midwives from a large area of central England to test the intervention in routine practice.

We achieved 77% for the primary outcome but only around 42% of women completed the end of pregnancy follow up questionnaires,

Although we assessed intervention fidelity, our data on the intervention group were incomplete, with only 65% of weight charts available.

For peer review only

# INTRODUCTION

In developed countries around 40-60% of women gain more weight while pregnant than the US Institute of Medicine (IOM) guidelines advise.[1-3] Excessive gestational weight gain is associated with adverse pregnancy outcomes and later obesity.[4-5] Although excessive gestational weight gain is common, no country has an evidence-based intervention to prevent it which can be used in routine care, and there is no global consensus about whether weighing during pregnancy prevents excessive gestational weight gain.[6] Most randomised controlled trials (RCTs) to date have focussed on specialist interventions for obese women who are pregnant, but most women who become pregnant are healthy or overweight, not obese. Pregnancy may be the time when weight control slips and preventative interventions are needed for these women to reduce long-term health risks and potential adverse effects on the infant.

Previous trials involving pregnant women have not found regular weighing either by maternity health professionals within antenatal care, or by women themselves, to be effective in reducing excessive gestational weight gain, although these trials have been small, and/or reported intervention contamination or experienced low adherence to the intervention.[7-10] Collectively this highlights the need for additional high quality trials to evaluate interventions that are embedded into routine clinical care. Whilst regular weighing during pregnancy and advice on optimal weight gain is part of standard antenatal care for pregnant women in many developed countries (e.g. USA, Canada, France), this is not the case in many other similar countries (e.g. Australia, New Zealand & Netherlands).[6] In England, the National Institute for Health and Care Excellence (NICE) do not recommend this because of a lack of evidence of effectiveness and concerns about the potential for psychological harm.[11] Thus we had the opportunity to test the effectiveness of introducing weighing into routine antenatal care in an environment where it is not the norm.

<u>Trial development and aims</u>



For peer review only - http://bmjopen.bmj.com/site/about/guidelines.xhtml

In preparation for the current study we conducted a feasibility RCT (POPS) to test the acceptability of an intervention where community midwives weighed women, set maximal gestational weight gain limits.[12] The recruitment rate was high at 94% demonstrating women were very keen to participate in the study. The feasibility trial also included two embedded qualitative studies (interviews) with both women and community midwives. Most women felt the intervention was useful in encouraging them to think about their weight and believed it should be part of routine antenatal care. The community midwives commented the intervention could be implemented within routine care without adding substantially to consultation length. Following our feasibility study our aim in the POPS2 trial was to investigate the effectiveness of a behavioural brief intervention based on target setting, routine antenatal weighing, and feedback in preventing excessive weight gain.[12-13] This was compared with usual maternity care.

## MATERIALAND METHODS

Trial design and population

POPS2 was an individually randomised clinical trial (RCT). The trial protocol has been published previously.[13]

Participant identification and recruitment

Participants

Pregnant women under the care of four maternity centres in England were recruited. Women received written information about the study and, if eligible were approached after their routine dating scan at 10-14 weeks gestation. Women were eligible if they were confirmed as having a singleton pregnancy with a body mass index (BMI) ≥18.5 kg/m$^2$ at recruitment, expected to receive community midwife (CMW) led care or shared care (midwife and consultant led care) at recruitment, were aged ≥18 years and between $10^{+0}$-$14^{+6}$ weeks gestation at recruitment. Women were not eligible if they were unable to understand English or provide



For peer review only - http://bmjopen.bmj.com/site/about/guidelines.xhtml

informed consent, attending a weight management programme, experiencing severe mental illness or dependent on illicit drugs or alcohol.

Randomisation and masking

The randomisation list was created by an independent statistician using nQuery Advisor version 7.0. Randomisation was stratified by BMI category at recruitment (healthy weight/overweight/obese) and recruitment site. Participants were individually randomised using random permuted blocks of mixed size (2, 4 or 6). The trial statistician remained blinded to group allocation until completion of analyses. Participants were allocated to the groups by a clinical trials unit telephone randomisation service. Allocation was revealed to researchers by calling the randomisation line.

Primary outcome

The primary outcome was the proportion of women who exceeded the IOM guidelines for healthy weight gain at 38 weeks gestation defined by their BMI-appropriate chart. The primary outcome was defined as the proportion of women whose weight was above the upper limit of the weight gain recommendation at the gestational age at which they were measured. Many women did not know their pre-pregnancy weight so for these women we assumed that they had gained a healthy amount of weight indicated for their gestational age in their BMI-appropriate IOM chart.

Secondary outcomes

Secondary weight-related outcomes were the proportion of women who were within the IOM guidelines for their early pregnancy BMI category at 38 weeks of pregnancy defined by their chart; proportion who were below healthy weight; weight gain (kilogram (kg) per week of pregnancy from baseline to end of pregnancy, defined as change in weight by gestational weeks and weight gain from baseline to 38 weeks gestation (kg). Other secondary outcomes were depression, anxiety and physical activity.[14-15] We also recorded pregnancy-related health outcomes, principally to contribute to future meta-analyses.



For peer review only

For peer review only - http://bmjopen.bmj.com/site/about/guidelines.xhtml

Baseline assessment and follow up of outcomes

All participants were weighed only in light clothing at baseline by the research team using calibrated scales and had their height measured. In England midwives see women at 38 weeks gestation and all participants were weighed then for the primary outcome. As a measure of intervention contamination, the usual care group were asked the question *"Did your midwife talk to you about your weight at your last two appointments?"*

Intervention

The intervention supplemented usual antenatal care and was based on self-regulation theory[16]. Self-regulation has been described as a process that has three distinct stages; self-monitoring, self-evaluation and self-reinforcement. Self-monitoring is a method of systematic self-observation, periodic measurement and recording of target behaviours with the goal of increasing self-awareness. The awareness fostered during self-monitoring is considered an essential initial step in promoting and sustaining behaviour change.

We aimed for the intervention to make minimal demands on midwives' time as this would be key to future implementation in routine care. The IOM guideline was the only one available for healthy pregnancy weight gain at the time of the study and we used it to set the limits for weight gain for the intervention group.[1] In UK antenatal care, there are typically eight antenatal consultations and we scheduled for the intervention to take place in each one. CMWs weighed women at each antenatal appointment using calibrated portable weighing scales. Midwives plotted women's weight on an IOM weight chart appropriate to a participant's BMI category at recruitment (Figure 1). The chart was attached to the woman's hand-held pregnancy notes and outlined a maximum weight gain limit for the next appointment. The published protocol explains how these maximum limits were set.[13] The goal was for weight gain to follow the midpoint line on the chart (Figure 1).

At subsequent appointments, midwives gave women feedback on weight gain in relation to the limit, set a new limit for the next appointment, and reinforced the value and importance of



For peer review only - http://bmjopen.bmj.com/site/about/guidelines.xhtml

healthy weight gain. Midwives never asked women to lose weight. Instead, midwives set targets to bring women back towards the centre line (Figure I). Midwives encouraged women to weigh themselves weekly and to record this on the chart, to calculate their weekly weight gain limits, and to check progress against the chart. Midwives offered brief advice about healthy eating and exercise in pregnancy.[17]

Training of community midwives

The research team trained midwives to deliver all components of the intervention as detailed in the published study protocol.[13] Mindful that only interventions requiring a short training course would ever be widely implemented in routine antenatal care, we designed a 60–70 minute module delivered in a group setting to community midwives. A training manual was also developed which included information on study eligibility criteria, recruitment procedures, the importance of adhering to protocol and not contaminating the usual care group. Information on the effects of weight gain during pregnancy, instructions about how to weigh and plot weight on the IOM weight chart and how to give feedback on the weight gain chart and example messages were also outlined. Explanation of how to set weight gain limits using the charts and examples of educational and motivational messages that should be given about gestational weight gain, diet and physical activity during pregnancy were also included. Midwives also practiced completing the weight gain charts using prepared case studies.

Usual care group

The usual care group received standard maternity care and no other intervention. As the intervention did not involve giving lifestyle advice, we did not ask community midwives to refrain from offering usual advice about diet and exercise early in pregnancy to the usual care group.

Patient involvement

This study was informed by a feasibility study where feedback from pregnant women and community midwives was integrated into this study. Feedback from a maternity public



For peer review only

For peer review only - http://bmjopen.bmj.com/site/about/guidelines.xhtml

involvement group (PRIME) was also incorporated in to the design of this study. Participants were not involved in study recruitment. Participants received a summary of the study.

Sample size

610 women (305 per group) was sufficient to detect a 15 percentage points difference between the groups (45% vs 60%) in the proportion who exceeded the IOM guideline for gestational weight gain with 90% power and 5% significance.[1-3] The sample size was inflated by 1.4 to allow for a midwife intra-cluster correlation coefficient of 0.036 estimated from our feasibility RCT and assumed that an average of 12 women would be under the care of each midwife and there would be 35 midwives.[12] The sample size included allowance for 20% loss to follow up. With these considerations, 610 participants (305 per group) were required and this would also be sufficient to detect 1.6kg group difference in mean weight at follow up (SD of weight change of 5.5kg and ICC=0.08 from feasibility RCT,[12] 90% power, 5% significance level.

Statistical Analysis

All analyses were performed using an intention to treat (ITT) approach, whereby participants were analysed according to randomisation group, with the following pre-defined exclusions: women who experienced pregnancy loss excluded from all analyses; women who experienced a preterm birth excluded from analyses of weight outcomes. The primary analysis, comparing the proportion exceeding the IOM guidelines between the groups was undertaken using generalised linear mixed modelling with imputation and the intervention effect presented as odds ratios (OR) with corresponding 95% confidence intervals (CI). We anticipated that the missingness mechanism for the majority of those with missing weights in the usual care group would be related to births taking place outside the due date and unrelated to their weight i.e. missing at random (MAR), therefore missing follow up weights were imputed using multiple imputation via PROC MI in SAS using five replications with group allocation, site, age, ethnicity, IMD quartile, BMI, baseline weight and gestation, as predictors. Weights were



For peer review only - http://bmjopen.bmj.com/site/about/guidelines.xhtml

considered missing if a self-reported weight was not available, or was not missing but measured before 37 weeks and delivery was not preterm. The primary analysis adjusted for BMI category and site as fixed effects and midwife as a random effect. A sub-group analysis assessing whether there were differences in treatment effect by BMI category was carried out by including a multiplicative interaction term in the modelling. We reasoned that both midwives and women might be more motivated to avoid excessive gain if women were already overweight.

The robustness of the results was examined with a sensitivity analysis of the primary outcome and included: complete case analysis; missing 38-week weights imputed with BMI category-specific mean weight; missing 38-week weights imputed with average weight within BMI category related IOM threshold. Secondary weight-related outcomes were compared using mixed modelling with multiple imputation and covariate adjustments as previously described. Linear mixed modelling was used to compare psychological health and physical activity at the end of pregnancy adjusting for baseline values of the outcome in addition to the covariates described above.

We also conducted per protocol analyses of the primary outcome. Adherence to the protocol was defined in two ways. First, we considered that women had followed the protocol if they recorded a weekly weight on at least 70% of occasions prior to delivery or 38 weeks gestation. Second, we assumed adherence if women had recorded at least five weekly weights, an approach taken by a similar study.[7] We considered midwives had followed the protocol if they set a correct weight gain target and subsequent weekly targets for women on 70% of a woman's appointments.

**RESULTS**

<u>Trial flow and characteristics of the population</u>

We approached 1,271 women and 816 were eligible (54.8%). Of these, 656 (75.8%) agreed to participate and were randomised, 329 to receive the intervention and 327 to usual care) (Figure 2). Baseline characteristics were similar between the trial groups (Table 1). 107



For peer review only - http://bmjopen.bmj.com/site/about/guidelines.xhtml

For peer review only

CMWS were trained to deliver the intervention. The first participant was randomised in November 2014 and follow up completed in December 2015.

**Table 1:** **Baseline characteristics by group**

| Characteristics | | Randomisation group | |
|---|---|---|---|
| | | **Intervention (N=329)** | **Usual care (N=327)** |
| Age (years) | mean (sd) | 29.4 (5.0) | 29.7 (5.2) |
| | range | 18.3 to 40.6 | 18.0 to 43.0 |
| Height (cm) | mean (sd) | 163.5 (6.5) | 163.3 (6.7) |
| Weight (kg) | mean (sd) | 69.3 (13.8) | 69.7 (13.5) |
| *BMI category* | | | |
| Healthy | | 161 (48.9) | 161 (49.2) |
| Overweight | | 106 (32.2) | 103 (31.5) |
| Obese | | 62 (18.8) | 63 (19.3) |
| BMI (kg/m$^2$) | mean (sd) | 25.9 (4.6) | 26.1 (4.8) |
| IMD quartile | 1 (least deprived) | 34/323 (10.5) | 31/325 (9.5) |
| | 2 | 60/323 (18.6) | 52/325 (16.0) |
| | 3 | 86/323 (26.6) | 96/325 (29.5) |
| | 4 (most deprived) | 143/323 (44.3) | 146/325 (44.9) |
| *Ethnicity* | | | |
| White | | 241/328 (73.5) | 238/327 (72.8) |
| Black Caribbean | | 5/328 (1.5) | 6/327 (1.8) |
| Black African | | 4/328 (1.2) | 5/327 (1.5) |
| Black Other | | 0/328 (0.0) | 1/327 (0.3) |
| Mixed | | 12/328 (3.7) | 7/327 (2.1) |
| Chinese | | 1/328 (0.3) | 3/327 (0.9) |
| Indian | | 10/328 (3.1) | 8/327 (2.5) |
| Pakistani | | 34/328 (10.4) | 39/327 (11.9) |
| Bangladeshi | | 4/328 (1.2) | 4/327 (1.2) |
| Other Asian | | 4/328 (1.2) | 2/327 (0.6) |
| Other | | 13/328 (4.0) | 14/327 (4.3) |
| *Marital status* | | | |
| Married | | 166/315 (52.7) | 195/318 (61.3) |
| Single (living alone) | | 48/315 (15.2) | 33/318 (10.4) |
| Single (living with spouse) | | 95/315 (30.8) | 86/318 (27.0) |
| Widowed | | 0/315 (0.0) | 1/318 (0.3) |
| Divorced/separated (living alone) | | 2/315 (0.6) | 2/318 (0.6) |
| Divorced/separated (living with spouse) | | 2/315 (0.6) | 1/318 (0.3) |
| *Employment status:* | | | |
| In Paid employment | | 218/319 (68.3) | 236/317 (74.5) |
| Student | | 15/319 (4.7) | 5/317 (1.6) |
| Self employed/freelance | | 21/319 (6.6) | 7/317 (2.2) |
| Looking after home/family | | 28/319 (8.8) | 24/317 (7.6) |
| Unemployed | | 35/319 (11.0) | 40/317 (12.6) |
| Sick/disabled | | 1/319 (0.3) | 1/317 (0.3) |
| Retired from paid work | | 0/319 (0.0) | 0/317 (0.0) |
| Other | | 1/319 (0.3) | 4/317 (1.3) |
| Smoking status: | | | |
| Current smoker | | 27/316 (8.5) | 20/317 (6.3) |
| Number of children | mean (sd) n | 0.6 (0.9) 313 | 0.8 (1.1) 315 |
| Attending weight loss programme | | 4/317 (1.3) | 5/317 (1.6) |

Figures are n (%) unless stated otherwise



For peer review only - http://bmjopen.bmj.com/site/about/guidelines.xhtml

For peer review only

There was no evidence of a difference in the proportion of women in the intervention and UC groups who gained excessive weight during pregnancy (intervention 27.6% versus usual care 28.9%, OR: 0.84, 95% CI: 0.53-0.33, p=0.46). Complete case analysis and different methods of imputation did not alter the results (Table 2). Sub-group analysis (Table 3) showed no evidence that the intervention effect differed by baseline BMI status (p=0.41).

On average women in the intervention group gained 10.3 kg and usual care gained 10.7kg between baseline and 38 weeks of pregnancy. There was no evidence of a difference in the change in weight (kg) during pregnancy (adjusted mean difference -0.42 kg 95% CI: -1.49-0.64) or the amount of weight gained per week of pregnancy between groups (adjusted mean difference -0.01 kg/week 95% CI: -0.038-0.018). There was no evidence of differences in the proportion of women in the groups who gained weight within the IOM guidelines (OR 0.92 95% CI: 0.63-1.32) or less than the minimum the IOM guidance (OR 1.26 95% CI: 0.86-1.83) for gestational weight (Table 3).

Women were doing less physical activity than is recommended for health in pregnancy, and by late pregnancy physical activity had declined, with no difference between groups: mean difference: -4.30 MET hrs/per/week 95% CI:-26.9-18.3 (Table 4).

There was no significant difference between groups in anxiety (mean difference -0.50 95% CI:-1.17-0.16) or depression scores (mean difference -0.59, 95% CI:-1.2-0.05) (Table 3).

No serious adverse events were reported. The numbers of pregnancy complications and adverse neonatal outcomes seemed similar in each group (supplementary Table 1). There was no evidence of intervention contamination in the usual care group.

We obtained 214 (65%) of the weight charts from participants' medical notes. Midwives plotted gestational weights and set weight targets in 57% and 50% respectively of scheduled antenatal appointments for the intervention group. Midwives recorded reminding women to weigh themselves weekly at 22% of scheduled appointments. Women in the intervention group weighed themselves on 34% of all weeks. A total of 50.9% (109/214) of women in the



For peer review only - http://bmjopen.bmj.com/site/about/guidelines.xhtml

intervention group weighed themselves five times or more, 15.9% (34/214) two-four times and 33.2% (71/214) once or less. In the per protocol analyses there was no evidence of a difference between the groups in the proportions who gained excessive gestation weight (supplementary Table 2).

For peer review only



For peer review only - http://bmjopen.bmj.com/site/about/guidelines.xhtml

**Table 2. Comparison of primary outcome (proportion exceeding BMI-related IOM guideline for weight gain during pregnancy) & subgroup analysis.**

| | Intervention | Usual care | | Intervention – Usual care | |
|---|---|---|---|---|---|
| | **n/N (%)** | **n/N (%)** | **Adjusted % difference (95%CI)** | **Adjusted odds ratio (95% CI)** | **P value** |
| **Primary analysis*** Proportion exceeding IOM guideline (multiple imputation of missing 38 week weights) | 81/305 (27.6) | 90/311 (28.9) | -3.5 (-17.8, 10.7) | 0.84 (0.53, 1.33) | 0.46 |
| | | | | | |
| **Sensitivity analysis of proportion exceeding IOM guideline** | | | | | |
| Complete case analysis$ | 51/215 (23.7) | 59/224 (26.3) | -4.8 (-19.8, 10.3) | 0.78 (0.48, 1.26) | 0.31 |
| Imputation with BMI category-specific mean weight# | 85/305 (27.9) | 91/311 (29.3) | -3.1 (-16.0, 9.8) | 0.86 (0.59, 1.26) | 0.44 |
| Imputation with average weight within BMI category related IOM threshold# | 87/305 (28.5) | 93/311 (29.9) | -3.1 (-16.0, 9.9) | 0.86 (0.59, 1.25) | 0.44 |
| | | | | | |
| **Subgroup** | **Intervention** | **Usual Care** | | | |
| **BMI category at recruitment** | **Number exceeding IOM guideline/N (%)+** | **Number exceeding IOM guideline/N (%)+** | **Adjusted odds ratio (95% CI)** | **P value (interaction)** | - |
| Healthy weight | 15/148 (10.3) | 22/161 (13.5) | 0.69 (0.22, 2.21 ) | 0.41 | - |
| Overweight | 38/95 (39.8) | 34/93 (36.6) | 1.11 (0.60, 2.04) | | |
| Obese | 31/62 (50.3) | 34/57 (59.6) | 0.69 (0.30, 1.58 ) | | |

Analysis adjusted by Site, BMI category and midwife (random effect). * includes objective and self-reported weights; missing weights imputed using multiple imputation; pooled estimates. $includes objective weights only #Includes objective and self-reported weight.+ includes objective and self-reported weights; missing weights imputed using multiple imputation; pooled estimates



For peer review only - http://bmjopen.bmj.com/site/about/guidelines.xhtml

**Table 3: Comparison of secondary outcome: proportion within or below BMI-related IOM guideline for weight gain during pregnancy.**

| | Intervention | Usual care | Intervention-usual care | | |
|---|---|---|---|---|---|
| | n/N (%) | n/N (%) | Adjusted % difference (95% CI) | Adjusted odds ratio (95% CI) | P value |
| Within IOM guideline$ | 96/305 (31.5) | 108/311 (34.6) | -2.0 (-14.6, 10.6) | 0.92 (0.63, 1.32) | 0.63 |
| Below IOM guideline$ | 125/305 (40.9) | 114/311 (36.5) | 4.9 (-7.4, 17.2) | 1.26 (0.862, 1.827) | 0.24 |

Analysis adjusted by Site, BMI category and midwife (random effect).
$ includes objective and self-reported weights; missing weights imputed using multiple imputation; pooled estimates.

For peer review only

**Table 4: Comparison of secondary outcomes.**

| | Intervention | | | Usual care | | | Intervention-Usual care | |
|---|---|---|---|---|---|---|---|---|
| | Baseline | 38 weeks | Change | baseline | 38 weeks | Change | | |
| | Mean (sd) n | Mean (sd) n | Mean (sd) n | Mean (sd) n | Mean (sd) n | Mean (sd) n | Adjusted Mean 38 weeks (95% CI)[b] | P value |
| **Weight (kg)[c]** | 71.21 (13.62) 305 | 81.49 (14.35) 305 | 10.28 (5.87) 305 | 71.06 (13.19) 311 | 81.79 (14.35) 311 | 10.73 (6.88) 311 | -0.42 (-1.49, 0.64) | 0.43 |
| **HADS: Anxiety** | 4.88 (3.50) 313 | 5.18 (3.09) 136 | 0.45 (2.82) 136 | 5.15 (3.28) 318 | 5.89 (3.58) 133 | 0.82 (3.33) 132 | -0.58 (-1.25, 0.08) | 0.08 |
| **HADS: Depression** | 3.29 (2.90) 313 | 3.93 (3.04) 136 | 0.75 (2.83) 136 | 3.49 (3.34) 318 | 4.56 (3.04) 133 | 1.29 (3.20) 132 | -0.60 (-1.24, 0.05) | 0.07 |
| **Total physical activity (Met/hrs/wk)** | 283.68 (144.52) 313 | 246.63 (104.97) 136 | -35.02 (115.94) 136 | 278.91 (158.50) 317 | 240.65 (115.26) 132 | -23.43 (117.05) 131 | -4.30 (-26.94, 18.34) | 0.71 |

[b]adjusted by baseline value, site, BMI category and midwife (random effect). [c] includes objective and self-reported weights; missing weights imputed using multiple imputation; pooled estimates



For peer review only - http://bmjopen.bmj.com/site/about/guidelines.xhtml

## DISCUSSION

There was no evidence that this intervention of weight gain limit setting, regular weighing and feedback delivered by CMWS as part of routine antenatal care was effective. There was however no evidence of psychological harm from the intervention. These findings contribute to the current and on-going debate about whether routine weighing should be re-introduced throughout pregnancy.

Pregnant women have reported that they expect to be weighed during pregnancy and feel it should be part of routine antenatal care.[12, 18] However, three previous trials have investigated the effectiveness of behavioural interventions based on regular weighing to prevent excessive gestational weight gain and none were effective.[7-10] In one, women were advised by a medical student to weigh themselves seven times during pregnancy and were given an IOM weight chart and a table for them to assess their own progress against the targets.[9] In a second, women spent half an hour discussing the importance of healthy weight gain with a research midwife and were encouraged to weigh themselves serially and given an overall weight target, which they were encouraged to discuss with a clinician.[8] However antenatal clinicians were not trained to intervene. In the third women were measured in antenatal clinics and their weight recorded; posters in the clinic were placed to raise women's motivation to stay within the IOM guidelines.[7] The intervention in the present trial was the most complete behavioural intervention to date, comprising both midwife training for routine weighing, setting weight gain limits and feedback, as well as individual advice to women to weigh themselves weekly.

The lack of effectiveness may be attributable to poor intervention delivery. Unlike previous trials, we recorded detailed information about intervention fidelity. In our feasibility trial most midwives commented that they felt the intervention was feasible taking on average about one to two minutes per appointment and it was not perceived as adding substantially to their workload. Midwives also commented that they liked the intervention because it was simple to do and provided them with a legitimate opportunity to raise the topic of gestational weight



For peer review only - http://bmjopen.bmj.com/site/about/guidelines.xhtml

gain. However here, the process evaluation showed only moderate fidelity by midwives in weighing women and setting a target, and little encouragement to women to weigh themselves at home. Only a small proportion of women weighed themselves every week through pregnancy.

Beyond pregnancy, among adults seeking to lose weight, adding regular self-weighing to behavioural weight loss programmes increases effectiveness.[19] The evidence from trials is supported by strong evidence that self-weighing is a key component of the behavioural repertoire of people who are successful at maintaining their weight.[20] However a programme based on self-weighing alone was only minimally effective.[19] We had expected that the greater engagement of women in their own health during pregnancy and concern for the health of their baby might make this a moment when regular weighing would prompt other self-regulatory controls and stimulate effective weight management. In the UK, NICE, recommends against weighing women routinely during antenatal care, and this practice is not part of antenatal care in many other countries, though it is routine in others. NICE noted the lack of evidence of benefit, but also expressed concerns that weighing may cause psychological harm. There was no evidence to indicate any health harms in this trial and other studies suggest that, far from increasing anxiety, it is welcomed by women.[12,18]

Previous research suggested that in many developed countries, the majority of women gain excess gestational weight. Only 28% of the usual care group did so here, not the 60% we assumed would in the sample size calculation. We can only speculate on why the proportion of women gaining excess weight was lower than expected in this trial. It may be due to contamination, with midwives intervening in some unspecified way among women in the usual care group, but we found no evidence of this in feedback from the usual care group and there were no weight charts in the notes of usual care women. Second, perhaps the trial enrolled women who were particularly weight conscious, as 25% of eligible women declined to participate. However, all of those who declined would need to have gained excess weight to reach the frequency of weight gain cited in other studies. One of the attractions of this kind of



For peer review only - http://bmjopen.bmj.com/site/about/guidelines.xhtml

programme is that it is scalable and well-suited to routine care, so that, if effective, it could be applied routinely in prevention in the way that few other interventions can. Future research will need to identify how to engage midwives and women more actively in the process of self-weighing, consider additional behavioural components or identify other interventions and test their effectiveness in this context.

This study has several strengths. Most (~76%) eligible women participated in the trial, meaning the results reflect the impact in the general population. A relatively large proportion of women were recruited from non-White ethnic groups (27%) and/or low socio-economic backgrounds (55%) who are often under-represented in trials. Weight was objectively assessed. Rather than recruit a small number of highly motivated and highly trained midwives, we trained over 100 midwives from a large area of central England to test the intervention in routine practice. To our knowledge this is the first trial where community midwives have delivered an intervention involving setting weight gain limits, regular weighing, encouraging weekly self-weighing, and providing feedback. Unlike trials testing similar interventions, we collected detailed process data on the fidelity of delivery of the intervention and women's adoption of the advice to weigh themselves.

Our findings should also be interpreted in light of some weakness. We estimated that we would follow-up 80% of participants for the primary outcome when calculating the sample size, and achieved 77%. However, only around 42% of women completed the end of pregnancy follow up questionnaires, despite reminders. Although we assessed fidelity, our data on the intervention group were incomplete, with only 65% of weight charts available. This was because some women experienced miscarriage, their notes were not available to the research team, they withdrew from the trial, or removed the charts from their notes. The proportion of women who gained excessive weight was markedly lower than predicted, 30% actual versus 60% predicted from the literature. The sample size was predicated on having 90% power to detect a 15% absolute risk reduction, a relative reduction in incidence of 25%. However, a 25% relative



For peer review only - http://bmjopen.bmj.com/site/about/guidelines.xhtml

reduction from 30% would imply a smaller absolute difference, thus reducing the power of the study below that originally envisaged, which means that a benefit of this treatment programme cannot be confidently ruled out.

**CONCLUSION**

We did not find evidence to support the value of setting a maximum weight gain limit, regular weighing, and feedback during pregnancy to prevent excessive gestational weight gain. The trial provides reassurance that weighing is not harmful, but in countries where regular weighing is part of usual maternity care, women should be advised that other strategies may be required to prevent excessive gestational weight gain.

For peer review only

**Figure legends**

Figure 1: Weight chart

Figure 2: Trial flow of participants

For peer review only



For peer review only - http://bmjopen.bmj.com/site/about/guidelines.xhtml

Author affiliations
[1]School of Sport, Exercise and Health Sciences, University of Loughborough, Epinal Way, Loughborough, Leicestershire, LE11 3TU

[2]Institute for Applied Health Research, College of Medical and Dental Sciences, University of Birmingham, Birmingham, B15 2TT

[3]Nuffield Department of Primary Care Health Sciences, University of Oxford, Radcliffe Observatory Quarter, Woodstock Road, Oxford, OX2 6GG

[4]Nuffield Department of Obstetrics and Gynaecology, University of Oxford, John Radcliffe Hospital, Headley Way, Oxford, OX3 9DU

[5]School of Social and Community Medicine, University of Bristol, Canynge Hall, 39 Whatley Road, Bristol, BS8 2PS

Ethics approval and consent to participate
Ethical approval was obtained from NRES Committee West Midlands – South Birmingham: 14/WM/1134, 02/10/14. All procedures performed in this study were in accordance with the ethical standards of the institutional review board, the American Psychological Association, and with the 1964 Helsinki Declaration. Written informed consent was obtained for all individual participants included in the study.

Patient consent for publication
Not applicable

Availability of the data and material
The datasets used and analysed during the current study are available from the corresponding author on reasonable request, after a period of two years from the date of this publication.

Competing interests
All authors declare that they have no conflict of interest. All procedures, including the informed consent process, were conducted in accordance with the ethical standards of the responsible committee on human experimentation (institutional and national) and with the Helsinki Declaration of 1975, as revised in 2000.

Funding
The National Institute of Health Research School for Primary Care Research part funded this study. NIHR CLAHRC-Oxford and NIHR CLAHRC-West Midlands also part funded this study. KJ is part funded by the National Institute for Health Research (NIHR) Collaboration for Leadership in Applied Health Research and Care (CLAHRC) West Midlands. SAJ and PA are NIHR Senior Investigators and supported by the Oxford NIHR Biomedical Research Centre and NIHR CLAHRC Oxford. The views expressed in this publication are not necessarily those of NHS, the NIHR or the Department of Health. The study was externally peer reviewed for scientific quality. The funders had no involvement in conducting the research.

Author contributions
AD conceived the original idea for the study with input from PA, KJ, SJ, AL and SC. AD wrote the protocol with contribution from the other authors. AR advised on statistical analyses and MU conducted the analyses. All authors had full access to the data, take responsibility for the integrity of the data and the accuracy of the data analysis, contributed to the interpretation of the results, and reviewed and approved the final manuscript. AD drafted the article and all other authors commented on this draft. AD is the guarantor.



For peer review only - http://bmjopen.bmj.com/site/about/guidelines.xhtml

For peer review only

Acknowledgements

We would like to thank the women who took part in this research. We would also like to thank the hospitals and community midwives who agreed to participate in this research; these were from Birmingham Women's NHS Foundation Trust; The Dudley Group NHS Foundation Trust; Oxford University Hospitals NHS Trust and South Warwickshire NHS Foundation Trust. We would also like to thank the PRIME PPI group at Birmingham Women's Hospital for their comments and feedback on the study.

For peer review only



For peer review only - http://bmjopen.bmj.com/site/about/guidelines.xhtml

## References

[1] Institute for Medicine. Weight gain in pregnancy: re-examining the guidelines. Washington, DC: The National Academy Press. 2007.

[2] Olson CM, Strawderman MS, Hinton PS, Pearson TA. Gestational weight gain and postpartum behaviors associated with weight change from early pregnancy to 1 y postpartum. *Int J Obes* 2003; **27**: 117–127.

[3] Cohen TR, Koski KG. Limiting excess weight gain in healthy pregnant women: importance of energy intake, physical activity, and adherence to gestational weight gain guidelines. *J Pregnancy* 2013; 787032: 1-6.

[4] Thangaratinam S, Rogozińska E, Jolly K, et al. Effects of interventions in pregnancy on maternal weight and obstetric outcomes: meta-analysis of randomised evidence. *BMJ* 2012; 344: e2088.

[5] Gunderson EP, Abrams B, Selvin S. The relative importance of gestational gain and maternal characteristics associated with the risk of becoming overweight after pregnancy. *Int J obesity Relat Metab Disord* 2000; **24**: 1660-1668.

[6] Scott C, Andersen CT, Valdez N, et al. No global consensus: a cross-sectional survey of maternal weight policies. *BMC Pregnancy Childbirth* 2014; **14**: 1-10.

[7] Brownfoot FC, Davey MA, Kornman L. Routine weighing to reduce excessive antenatal weight gain: a randomised controlled trial. *BJOG* 2016; **123**: 254-261.

[8] McCarthy EA, Walker SP, Ugoni A, Lappas M, Leong O, Shub A. Self-weighing and simple dietary advice for overweight and obese pregnant women to reduce obstetric complications without impact on quality of life: a randomised controlled trial. *BJOG* 2016; **123**: 965-73.

[9] Jeffries K, Shub A, Walker SP, Hiscock R, Permezel M. Reducing excessive weight gain in pregnancy: A randomised controlled trial. *Med J Aus* 2009; **191**: 429-433.

[10] Fealy SM, Taylor RM, Foureur M, et al. Weighing as a stand-alone intervention does not reduce excessive gestational weight gain compared to routine antenatal care: systematic review



For peer review only

and meta-analysis of randomised controlled trials. *BMC Pregnancy and Childbirth* 2017) **17**:36.

[11] National Institute for Clinical Excellence. Public Health Guidance 18: Weight Management Before, During and After Pregnancy: Public Health Guidance Scope 2010. London: NICE. Accessed at https://www.nice.org.uk/guidance/ph27 (Accessed 7th February 2018).

[12] Daley AJ, Jolly K, Jebb SA, et al. Feasibility and acceptability of regular weighing, setting weight gain limits and providing feedback by community midwives to prevent excess weight gain during pregnancy: randomised controlled trial and qualitative study. *BMC Public Health* 2015; **2**: 35.

[13] Daley AJ, Jolly K, Jebb SA, et al. Effectiveness of regular weighing, weight target setting and feedback by community midwives within routine antenatal care in preventing excessive gestational weight gain: randomised controlled trial. *BMC Obesity (study protocol)* 2016; **3**: 7.

[14] Zigmond AS, Snaith RP. The Hospital Anxiety and Depression Scale. *Acta Psychiatr Scan.* 1983; **67**: 361-70.

[15] Chasan-Taber L, Schmidt MD, Roberts DE, Hosmer D, Markenson G, Freedson PS. Development and validation of a pregnancy physical activity questionnaire. *Med Sci Sport Exer* 2004; **36**: 1750-1760.

[16] Kanfer FH. Self-monitoring: methodological limitations & clinical applications. *J Consult Clin Appli* 1970; **35**: 148–158.

[17] Royal College for Obstetrics and Gynaecology. Recreational exercise and pregnancy: information for you. 2006. London. https://www.rcog.org.uk/globalassets/documents/patients/patient-information-leaflets/pregnancy/recreational-exercise-and-pregnancy.pdf. (Accessed 7th Feb 2018).



For peer review only - http://bmjopen.bmj.com/site/about/guidelines.xhtml

[18] Allen-Walker V, Mullaney L, Turner MJ, et al. How do women feel about being weighed during pregnancy: A qualitative exploration of the opinions and experiences of postnatal women. *Midwifery in press* 2017; **49**: 95-101.

[19] Madigan CD, Daley AJ, Lewis AL, Aveyard P, Jolly K. Is self-weighing an effective tool for weight loss: A systematic literature review and meta-analysis. *I J Behav Nutri Phys Activ* 2015; **12**: 104.

[20] Wing RR, Tate DF, Gorin AA, Raynor HA, Fava JL. A self-regulation program for maintenance of weight loss. *The N Engl J Med* 2006; **355**: 1563-1571.

For peer review only



For peer review only - http://bmjopen.bmj.com/site/about/guidelines.xhtml

**Figure 1. Weight chart.**

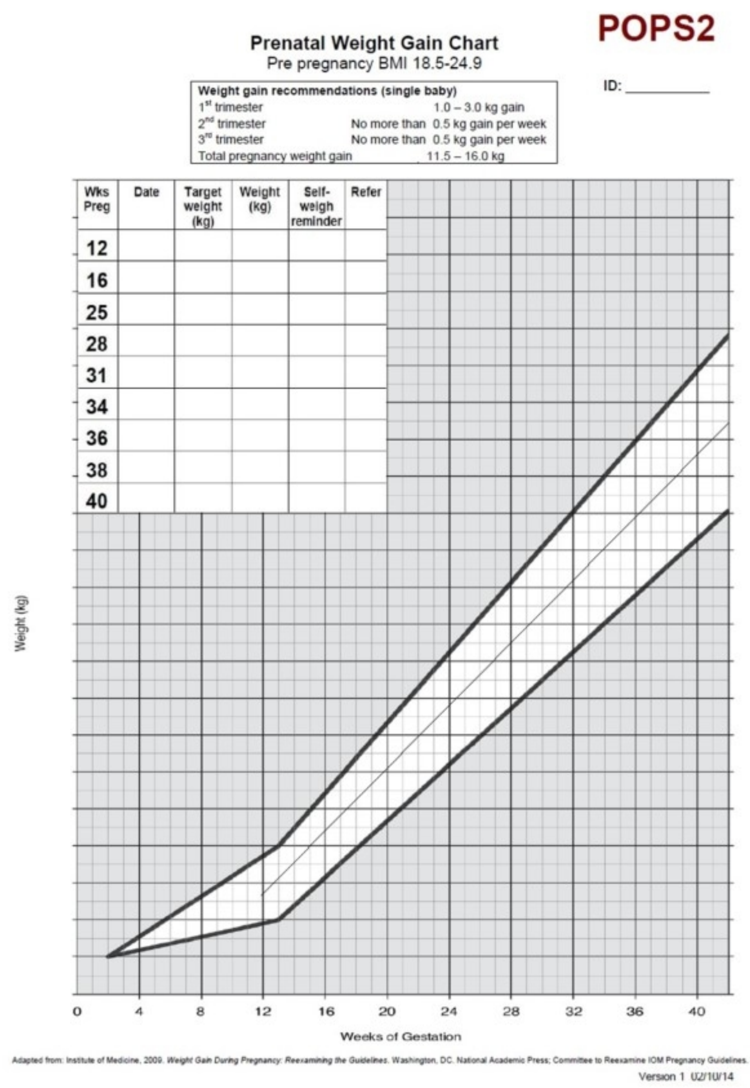


Figure 1: Weight chart

188x245mm (96 x 96 DPI)

**Figure 2: Trial flow**

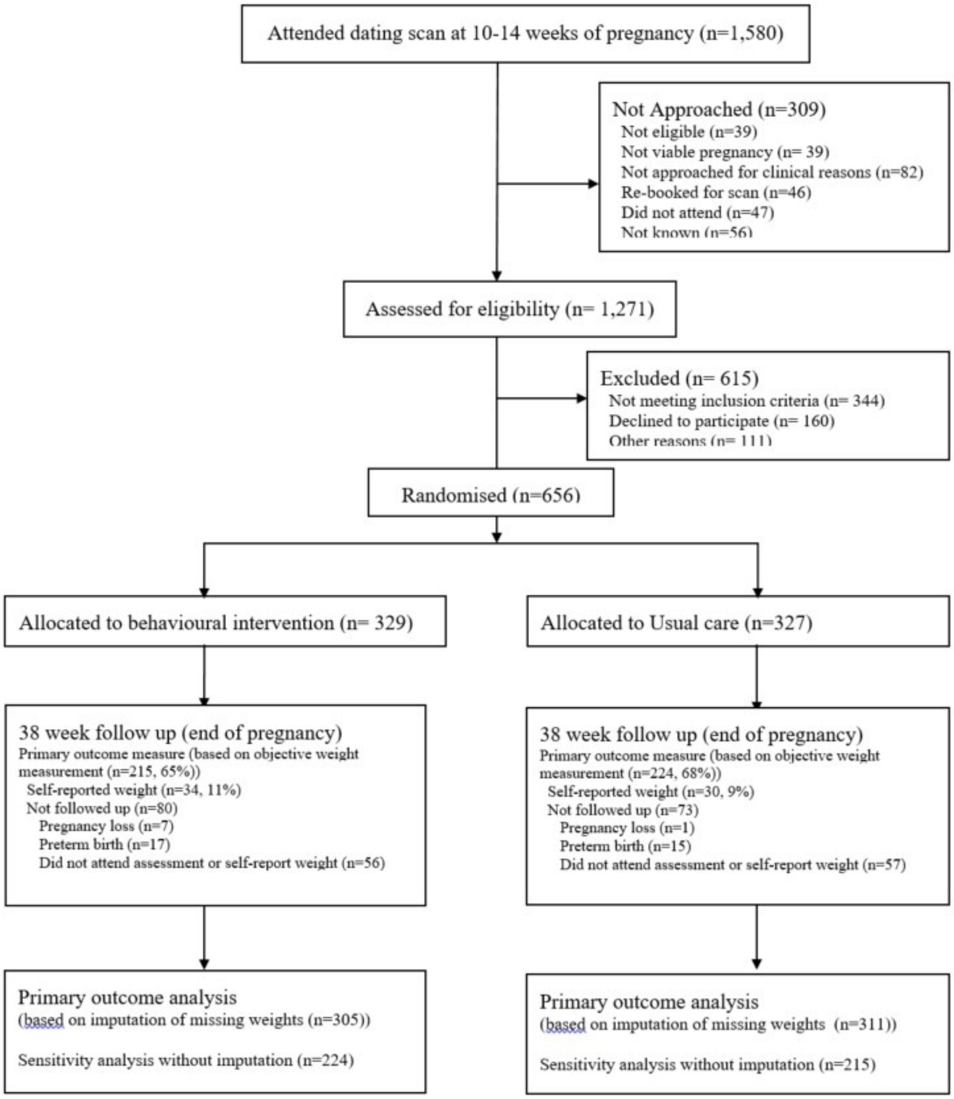


Figure 2: Trial flow

200x239mm (96 x 96 DPI)

**Supplementary Table 1: Maternal and neonatal complications by group**

| | Intervention | Usual care |
|---|---|---|
| **Mother:** | N (%) | N (%) |
| Caesarean section | 68/304 (22.4) | 69/302 (22.8) |
| Length of inpatient stay mean (sd)<br>median (IQR) | 1.7 (1.5) 305<br>1.0 (1.0 to 2.0) | 1.7 (1.7) 306<br>1.0 (1.0 to 2.0) |
| Maternal ICU admission | n/a | n/a |
| Preeclampsia | 6/315 (1.9) | 8/317 (2.5) |
| Pregnancy induced hypertension | n/a | n/a |
| Gestational diabetes | 12/315 (3.8) | 17/317 (5.4) |
| Maternal sepsis | n/a | n/a |
| Preterm delivery | 17/304 (5.6) | 14/302 (4.6) |
| Miscarriage | 5/317 (1.6) | 1/316 (0.3) |
| Stillbirth | 0/316 (0) | 1/316 (0.3) |
| Shoulder dystocia | 3/312 (1.0) | 2/314 (0.6) |
| **Baby:** | | |
| Treatment for jaundice | 34/312 (10.9) | 27/314 (8.6) |
| Low Apgar score (<7) at 1 min<br>Low apgar score (<7) at 5 mins | n/a<br>4/254 (1.6) | n/a<br>1/249 (0.4) |
| Admission to NICU | 26/261 (10.0) | 21/262 (8.0) |
| Neonatal death | n/a | n/a |
| Neonatal sepsis | n/a | n/a |
| Gestational age (wks) mean (sd)<br>median (IQR) | 39.2 (2.1) 304<br>40 (39-40) | 39.3 (1.6) 302<br>40 (38-40) |
| Birth weight (g) mean (sd)<br>median (IQR) | 3348.6 (567.1) 304<br>3373.5 (3060-3665) | 3401.8 (550.7) 301<br>3460 (3040-3745) |

For peer review only

Supplementary Table 2: Per-protocol analysis

| | Intervention | Usual care | Intervention-Usual care | | |
|---|---|---|---|---|---|
| | **Number exceeding IOM guideline/N$(%)** | **Number exceeding IOM guideline/N$(%)** | **% (95% CI)*** | **Adjusted odds ratio (95% CI)*** | **P value** |
| Women recorded weight weekly >70% of time during pregnancy | 7/46 (15.2) | 67/254 (26.4) | -9.2 (-26.3, 8.0) | 0.58 (0.23, 1.47) | 0.25 |
| Midwife overall accuracy for >70% appointments | 8/42 (19.0) | 67/254 (26.4) | -5.9 (-28.0, 16.2) | 0.71 (0.27, 1.88) | 0.49 |
| Women weighing themselves 5 or more times during pregnancy | 18/95 (18.9) | 67/254 (26.4) | -7.0 (-23.2, 9.2) | 0.68 (0.35, 1.33) | 0.26 |

$ Includes objective and self-reported weights
*adjusted by Site, BMI category and midwife (random effect)

For peer review only

# CONSORT 2010 checklist of information to include when reporting a randomised trial*

| Section/Topic | Item No | Checklist item | Reported on page No |
|---|---|---|---|
| **Title and abstract** | | | |
| | 1a | Identification as a randomised trial in the title | Title page |
| | 1b | Structured summary of trial design, methods, results, and conclusions (for specific guidance see CONSORT for abstracts) | 1 |
| **Introduction** | | | |
| Background and objectives | 2a | Scientific background and explanation of rationale | 3-4 |
| | 2b | Specific objectives or hypotheses | 4 |
| **Methods** | | | |
| Trial design | 3a | Description of trial design (such as parallel, factorial) including allocation ratio | 5 |
| | 3b | Important changes to methods after trial commencement (such as eligibility criteria), with reasons | N/A |
| Participants | 4a | Eligibility criteria for participants | 5-6 |
| | 4b | Settings and locations where the data were collected | 5 |
| Interventions | 5 | The interventions for each group with sufficient details to allow replication, including how and when they were actually administered | 7-8 |
| Outcomes | 6a | Completely defined pre-specified primary and secondary outcome measures, including how and when they were assessed | 6-7 |
| | 6b | Any changes to trial outcomes after the trial commenced, with reasons | N/A |
| Sample size | 7a | How sample size was determined | 9 |
| | 7b | When applicable, explanation of any interim analyses and stopping guidelines | N.A |
| Randomisation: | | | |
| Sequence generation | 8a | Method used to generate the random allocation sequence | 6 |
| | 8b | Type of randomisation; details of any restriction (such as blocking and block size) | 6 |
| Allocation concealment mechanism | 9 | Mechanism used to implement the random allocation sequence (such as sequentially numbered containers), describing any steps taken to conceal the sequence until interventions were assigned | 6 |
| Implementation | 10 | Who generated the random allocation sequence, who enrolled participants, and who assigned participants to interventions | 6 |
| Blinding | 11a | If done, who was blinded after assignment to interventions (for example, participants, care providers, those | N/A |

For peer review only

| | | | |
|---|---|---|---|
| | | assessing outcomes) and how | |
| | 11b | If relevant, description of the similarity of interventions | N.A |
| Statistical methods | 12a | Statistical methods used to compare groups for primary and secondary outcomes | 9-10 |
| | 12b | Methods for additional analyses, such as subgroup analyses and adjusted analyses | 9-10 |
| **Results** | | | |
| Participant flow (a diagram is strongly recommended) | 13a | For each group, the numbers of participants who were randomly assigned, received intended treatment, and were analysed for the primary outcome | 11 |
| | 13b | For each group, losses and exclusions after randomisation, together with reasons | 11 |
| Recruitment | 14a | Dates defining the periods of recruitment and follow-up | 11 |
| | 14b | Why the trial ended or was stopped | N/A |
| Baseline data | 15 | A table showing baseline demographic and clinical characteristics for each group | 19 |
| Numbers analysed | 16 | For each group, number of participants (denominator) included in each analysis and whether the analysis was by original assigned groups | 12-13 |
| Outcomes and estimation | 17a | For each primary and secondary outcome, results for each group, and the estimated effect size and its precision (such as 95% confidence interval) | 12-13 |
| | 17b | For binary outcomes, presentation of both absolute and relative effect sizes is recommended | 12-13 |
| Ancillary analyses | 18 | Results of any other analyses performed, including subgroup analyses and adjusted analyses, distinguishing pre-specified from exploratory | 12-13 |
| Harms | 19 | All important harms or unintended effects in each group (for specific guidance see CONSORT for harms) | 13 |
| **Discussion** | | | |
| Limitations | 20 | Trial limitations, addressing sources of potential bias, imprecision, and, if relevant, multiplicity of analyses | 12 |
| Generalisability | 21 | Generalisability (external validity, applicability) of the trial findings | 10-12 |
| Interpretation | 22 | Interpretation consistent with results, balancing benefits and harms, and considering other relevant evidence | 10-12 |
| **Other information** | | | |
| Registration | 23 | Registration number and name of trial registry | 1 |
| Protocol | 24 | Where the full trial protocol can be accessed, if available | 5 |
| Funding | 25 | Sources of funding and other support (such as supply of drugs), role of funders | 15 |

*We strongly recommend reading this statement in conjunction with the CONSORT 2010 Explanation and Elaboration for important clarifications on all the items. If relevant, we also recommend reading CONSORT extensions for cluster randomised trials, non-inferiority and equivalence trials, non-pharmacological treatments, herbal interventions, and pragmatic trials. Additional extensions are forthcoming: for those and for up to date references relevant to this checklist, see www.consort-statement.org.


For peer review only - http://bmjopen.bmj.com/site/about/guidelines.xhtml

BMJ Open

# Effectiveness of a behavioural intervention involving regular weighing and feedback by community midwives within routine antenatal care to prevent excessive gestational weight gain: POPS2 randomised controlled trial



| | |
|---|---|
| Journal: | *BMJ Open* |
| Manuscript ID | bmjopen-2019-030174.R1 |
| Article Type: | Research |
| Date Submitted by the Author: | 07-Jul-2019 |



| | |
|---|---|
| Complete List of Authors: | Daley, Amanda; Loughborough University, School of Sport, Exercise and Health Sciences<br>Jolly, Kate; University of Birmingham<br>Jebb, Susan; Univerof Oxford, Primary Care Health Sciences<br>Roalfe, Andrea; Oxford University<br>Mackilllop, Lucy; John Radcliffe Hospital<br>Lewis, Amanda; University of Bristol<br>Clifford, Sue; University of Birmingham College of Medical and Dental Sciences<br>Usman, Muhammad; University Hospitals of Leicester NHS Trust<br>Ohadike, Corah; Oxford University<br>Kenyon, Sara; University of Birmingham, Public Health, Epidemiology and Biostatistics<br>MacArthur, Christine; University of Birmingham, Public Health<br>Aveyard, Paul; University of Oxford, Primary Care Health Sciences |



| | |
|---|---|
| <b>Primary Subject Heading</b>: | Obstetrics and gynaecology |
| Secondary Subject Heading: | Nutrition and metabolism, Public health |
| Keywords: | pregnancy, weight, weighing, pregnancy, weighing, midwives, weight |
| | |


SCHOLARONE™
Manuscripts

# Effectiveness of a behavioural intervention involving regular weighing and feedback by community midwives within routine antenatal care to prevent excessive gestational weight gain: POPS2 randomised controlled trial

**Short Title: Routine weighing during pregnancy by community midwives**

Amanda J Daley[1] PhD, a.daley@lboro.ac.uk
Kate Jolly[2] PhD, c.b.jolly@bham.ac.uk
Susan A Jebb[3] PhD, susan.jebb@phc.ox.ac.uk
Andrea K Roalfe[3] MSc, andrea.roalfe@phc.ox.ac.uk
Lucy Mackillop[4] MA (Oxon.), Lucy.MacKillop@ouh.nhs.uk
Amanda L Lewis[5] PhD, amanda.lewis@bristol.ac.uk
Sue J Clifford[2] BSc, s.clifford@bham.ac.uk
Muhammad Usman[2] MSc, musman.bstat@gmail.com
Corah O Ohadike[4] BMBS, corah.ohadike@wrh.ox.ac.uk
Sara Kenyon[2] PhD, s.kenyon@bham.ac.uk
Christine MacArthur[2] PhD, c.macarthur@bham.ac.uk
Paul Aveyard[3] PhD, paul.aveyard@phc.ox.ac.uk

[1]School of Sport, Exercise and Health Sciences, University of Loughborough, Epinal Way, Loughborough, Leicestershire, LE11 3TU

[2]Institute of Applied Health Research, College of Medical and Dental Sciences, University of Birmingham, Birmingham, B15 2TT

[3]Nuffield Department of Primary Care Health Sciences, University of Oxford, Radcliffe Observatory Quarter, Woodstock Road, Oxford, OX2 6GG

[4]Nuffield Department of Women's and Reproductive Health, University of Oxford, John Radcliffe Hospital, Headley Way, Oxford, OX3 9DU

[5]Population Health Sciences, Bristol Medical School, University of Bristol, Canynge Hall, 39 Whatley Road, Bristol, BS8 2PS

**Corresponding author**
Professor Amanda Daley
School of Sport, Exercise and Health Sciences
University of Loughborough
Epinal Way
Loughborough
Leicestershire, LE11 3TU
a.daley@lboro.ac.uk

Word count: 3,813

For peer review only

**Abstract**

**Objectives:** To assess the effectiveness of a brief behavioural intervention based on routine antenatal weighing to prevent excessive gestational weight gain (defined by United States Institute of Medicine).

**Design:** Randomised controlled trial.

**Setting:** Antenatal clinical in England.

**Participants:** Women between $10^{+0}$ and $14^{+6}$ weeks gestation, not requiring specialist obstetric care,

**Interventions:** Participants were randomised to usual antenatal care or usual care plus the intervention. The intervention involved community midwives weighing women at antenatal appointments, setting maximum weight gain limits between appointments and providing brief feedback. Women were encouraged to monitor and record their own weight weekly to assess their progress against the maximum limits set by their midwife. The comparator was usual maternity care.

**Primary and secondary outcome measures:** Excessive gestation weight gain, depression, anxiety and physical activity.

**Results** 656 women from four maternity centres were recruited; 329 randomised to the intervention group and 327 to usual care. We found no evidence that the intervention decreased excessive gestational weight gain. At 38 weeks gestation the proportions gaining excessive gestational weight were 27.6% (81/305) versus 28.9% (90/311) (adjusted odds ratio 0.84, 95% CI: 0.53-1.33) in the intervention and usual care group respectively. There were no significant differences between the groups in anxiety and depression scores (anxiety: adjusted mean -0.50, 95% CI:-1.17-0.16; depression: adjusted mean -0.59, 95% CI:-1.23-0.05). There were no significant differences in physical activity scores between the groups.

**Conclusions**: A behavioural intervention delivered by community midwives involving routine weighing throughout pregnancy, setting maximum weight gain targets, and encouraging women



For peer review only - http://bmjopen.bmj.com/site/about/guidelines.xhtml
For peer review only

to weigh themselves each week to check progress did not prevent excessive gestational weight gain. There was no evidence of psychological harm.

**Trial registration:** Current Controlled Trials registry number ISRCTN 67427351. 29th October 2014. http://www.isrctn.com/ISRCTN67427351.

**Key words**: pregnancy, weighing, midwives, weight

**Strength and limitations of this study**

Most (80%) eligible women participated in the trial, meaning the results reflect the impact in the general population.

A relatively large proportion of women were recruited from non-White ethnic groups and/or low socio-economic backgrounds.

Weight was objectively assessed and we trained over 100 midwives from a large area of central England to test the intervention in routine practice.

We achieved 77% follow up for the primary outcome but only around 42% of women completed the end of pregnancy follow up questionnaires,

Although we assessed intervention fidelity, our data on the intervention group were incomplete, with only 65% of weight charts available.

For peer review only

## INTRODUCTION

In developed countries around 40-60% of women gain more weight while pregnant than the US Institute of Medicine (IOM) guidelines advise.[1-3] Excessive gestational weight gain is associated with adverse pregnancy outcomes and later obesity.[4-5] Although excessive gestational weight gain is common, no country has an evidence-based intervention to prevent it which can be used in routine care, and there is no global consensus about whether weighing during pregnancy prevents excessive gestational weight gain.[6] Most randomised controlled trials (RCTs) to date have focussed on specialist interventions for obese women who are pregnant, but most women who become pregnant are healthy or overweight, not obese. Pregnancy may be the time when weight control slips and preventative interventions are needed for these women to reduce long-term health risks and potential adverse effects on the infant.

Previous trials involving pregnant women have not found regular weighing either by maternity health professionals within antenatal care, or by women themselves, to be effective in reducing excessive gestational weight gain, although these trials have been small, and/or reported intervention contamination or experienced low adherence to the intervention.[7-10] Collectively this highlights the need for additional high quality trials to evaluate interventions that are embedded into routine clinical care. Whilst regular weighing during pregnancy and advice on optimal weight gain is part of standard antenatal care for pregnant women in many developed countries (e.g. USA, Canada, France), this is not the case in many other similar countries (e.g. Australia, New Zealand and Netherlands).[6] In England, the National Institute for Health and Care Excellence (NICE) do not recommend this because of a lack of evidence of effectiveness and concerns about the potential for psychological harm.[11] Thus we had the opportunity to test the effectiveness of introducing weighing into routine antenatal care in an environment where it is not the norm.

<u>Trial development and aims</u>

For peer review only

In preparation for the current study we conducted a feasibility RCT (POPS) to test the acceptability of an intervention where community midwives weighed women, set maximal gestational weight gain limits.[12] The recruitment rate was high at 94% demonstrating women were very keen to participate in the study. The feasibility trial also included two embedded qualitative studies (interviews) with both women and community midwives. Most women felt the intervention was useful in encouraging them to think about their weight and believed it should be part of routine antenatal care. The community midwives commented the intervention could be implemented within routine care without adding substantially to consultation length. Following our feasibility study our aim in this trial (POPS2) was to investigate the effectiveness of a behavioural brief intervention based on target setting, routine antenatal weighing, and feedback in preventing excessive weight gain.[12-13] This was compared with usual maternity care.

## MATERIALS AND METHODS

<u>Trial design and population</u>

POPS2 was randomised clinical trial (RCT) with individual randomisation. The trial protocol has been published previously.[13]

<u>Participant identification and recruitment</u>

<u>Participants</u>

Pregnant women under the care of four maternity centres in England were recruited. Women received written information about the study and, if eligible were approached after their routine dating scan at 10-14 weeks gestation. Women were eligible if they were confirmed as having a singleton pregnancy with a body mass index (BMI) ≥18.5 kg/m$^2$ at recruitment, expected to receive community midwife led care or shared care (midwife and consultant led care) at recruitment, were aged ≥18 years and between $10^{+0}$ to $14^{+6}$ weeks gestation at recruitment. Women were not eligible if they were unable to understand English or provide informed consent, attending a weight management programme, experiencing severe mental illness or dependent on illicit drugs or alcohol.



For peer review only - http://bmjopen.bmj.com/site/about/guidelines.xhtml

Randomisation and masking

The randomisation list was created by an independent statistician using nQuery Advisor version 7.0. Randomisation was stratified by BMI category at recruitment (healthy weight/overweight/obese) and recruitment site. Participants were individually randomised using random permuted blocks of mixed size (2, 4 or 6). Due to the nature of the intervention it was not possible to blind participants or community midwives to the intervention. The trial statistician remained blinded to group allocation until completion of analyses. Participants were allocated to the groups by a clinical trials unit telephone randomisation service. Allocation was revealed to researchers by calling the randomisation line.

Primary outcome

The primary outcome was the proportion of women who exceeded the upper limit of the IOM guidelines for healthy weight gain at 38 weeks gestation defined by their BMI-appropriate weight chart. Weight gain at 38 weeks was calculated as before 37 weeks were classed as pre-term and excluded from weight related analyses.

Secondary outcomes

Secondary weight-related outcomes were the proportion of women who were within the IOM guidelines for their early pregnancy BMI category at 38 weeks of pregnancy defined by their chart; proportion who were below the IOM guidance for healthy weight, weight gain (kilogram (kg) per week of pregnancy from baseline to end of pregnancy, defined as change in weight by gestational weeks and weight gain from baseline to 38 weeks gestation (kg). Other secondary outcomes were change in depression and anxiety between baseline and 38 weeks measured by the Hospital Anxiety and Depression Scale (HADS), physical activity measured by the Physical Activity in Pregnancy Questionnaire and diet quality measured by the Southampton Food Frequency Questionnaire.[14-16] We also recorded pregnancy-related health outcomes, principally to contribute to future meta-analyses.

Baseline assessment and follow up of outcomes



For peer review only - http://bmjopen.bmj.com/site/about/guidelines.xhtml

All participants were weighed only in light clothing at baseline by the research team using calibrated scales and had their height measured. In England midwives see women at 38 weeks gestation and all participants were weighed then for the primary outcome. As a measure of intervention contamination, the usual care group were asked the question *"Did your midwife talk to you about your weight at your last two appointments?"*

Intervention

The intervention supplemented usual antenatal care and was based on self-regulation theory.[17] Self-regulation has been described as a process that has three distinct stages; self-monitoring, self-evaluation and self-reinforcement. Self-monitoring is a method of systematic self-observation, periodic measurement and recording of target behaviours with the goal of increasing self-awareness. The awareness fostered during self-monitoring is considered an essential initial step in promoting and sustaining behaviour change.

We aimed for the intervention to make minimal demands on midwives' time as this would be key to future implementation in routine care. The IOM guideline was the only one available for healthy pregnancy weight gain at the time of the study and we used it to set the limits for weight gain for the intervention group.[1] In UK antenatal care, there are typically eight antenatal consultations and we scheduled for the intervention to take place in each one. Midwives weighed women at each antenatal appointment using calibrated portable weighing scales. Midwives plotted women's weight on an IOM weight chart appropriate to a participant's BMI category at recruitment (Figure 1). The chart was attached to the woman's hand-held pregnancy notes and outlined a maximum weight gain limit for the next appointment. The published protocol explains how these maximum limits were set.[13] The goal was for weight gain to follow the midpoint line on the chart (Figure 1).

At subsequent appointments, midwives gave women feedback on weight gain in relation to the limit, set a new limit for the next appointment, and reinforced the value and importance of healthy weight gain. Midwives never asked women to lose weight. Instead, midwives set targets



For peer review only - http://bmjopen.bmj.com/site/about/guidelines.xhtml

to bring women back towards the centre line (Figure I). Midwives encouraged women to weigh themselves weekly and to record this on the chart, to calculate their weekly weight gain limits, and to check progress against the chart. Midwives offered brief advice about healthy eating and exercise in pregnancy.[18]

Training of community midwives

The research team trained midwives to deliver all components of the intervention as detailed in the published study protocol.[13] Mindful that only interventions requiring a short training course would ever be widely implemented in routine antenatal care, we designed a 60–70 minute module delivered in a group setting to community midwives. A training manual was also developed which included information on study eligibility criteria, recruitment procedures, the importance of adhering to protocol and not contaminating the usual care group. Information on the effects of weight gain during pregnancy, instructions about how to weigh and plot weight on the IOM weight chart and how to give feedback on the weight gain chart and example messages were also outlined. Explanation of how to set weight gain limits using the charts and examples of educational and motivational messages that should be given about gestational weight gain, diet and physical activity during pregnancy were also included. Midwives also practiced completing the weight gain charts using prepared case studies.

Usual care group

The usual care group received standard maternity care and no other intervention. As the intervention did not involve giving lifestyle advice, we did not ask community midwives to refrain from offering usual advice about diet and exercise early in pregnancy to the usual care group.

Patient and public involvement in this research

Feedback from pregnant women and community midwives that was obtained by a previous feasibility study, was integrated into the design and conduct of this study. In addition, feedback from a maternity patient and public involvement group at local hospital (PRIME) was also



For peer review only - http://bmjopen.bmj.com/site/about/guidelines.xhtml

incorporated in to the design of this study. Participants in this study were not involved in the study recruitment processes. Study participants received a summary of the study results once it was completed.

Sample size

610 women (305 per group) was sufficient to detect a 15 percentage points difference between the groups (45% vs 60%) in the proportion who exceeded the IOM guideline for gestational weight gain with 90% power and 5% significance.[1-3] The sample size included allowance for 20% loss to follow up. This sample size would also be sufficient to detect 1.6kg group difference in mean weight gain at follow up (SD of weight change of 5.5kg from our feasibility RCT,[12] 90% power, 5% significance level. The sample size was not inflated for clustering by midwife because clustering of this nature does not inflate the type 1 error rate as described elsewhere.[19]

Statistical Analysis

All analyses were performed using an intention to treat (ITT) approach, whereby participants were analysed according to randomisation group, with the following pre-defined exclusions: women who experienced pregnancy loss excluded from all analyses; women who experienced a preterm birth excluded from analyses of weight outcomes. The primary analysis, comparing the proportion exceeding the IOM guidelines between the groups was undertaken using generalised linear mixed modelling with imputation and the intervention effect presented as odds ratios (OR) with corresponding 95% confidence intervals (CI). We anticipated that the missingness mechanism for the majority of those with missing weights in the usual care group would be related to births taking place outside the due date and unrelated to their weight i.e. missing at random (MAR), therefore missing follow up weights were imputed using multiple imputation via PROC MI in SAS using five replications with group allocation, site, age, ethnicity, IMD quartile, BMI, baseline weight and final weight and gestation, as predictors. Weights were considered missing if a self-reported weight was not available, or was not missing but measured before 37 weeks and delivery was not preterm. The primary analysis adjusted for BMI category and site as fixed effects and midwife as a random effect. A sub-group analysis



For peer review only - http://bmjopen.bmj.com/site/about/guidelines.xhtml

assessing whether there were differences in treatment effect by BMI category was carried out by including a multiplicative interaction term in the modelling. We reasoned that both midwives and women might be more motivated to avoid excessive gain if women were already overweight.

The robustness of the results was examined with a sensitivity analysis of the primary outcome and included: complete case analysis; missing 38-week weights imputed with BMI category-specific mean weight; missing 38-week weights imputed with average weight within BMI category related IOM threshold. Secondary weight-related outcomes were compared using mixed modelling with multiple imputation and adjustments for BMI category, site and midwife as previously described. Linear mixed modelling was used to compare psychological health and physical activity at the end of pregnancy adjusting for baseline values of the outcome in addition to BMI category, site and midwife..

We also conducted per protocol analyses of the primary outcome. Adherence to the protocol was defined in two ways. First, we considered that women had followed the protocol if they recorded a weekly weight on at least 70% of occasions prior to delivery or 38 weeks gestation. Second, we assumed adherence if women had recorded at least five weekly weights, an approach taken by a similar study.[7] We considered midwives had followed the protocol if they set a correct weight gain target and subsequent weekly targets for women on 70% of a woman's appointments.

## RESULTS

Trial flow and characteristics of the population

We approached 1,271 women and 816 were eligible (64.2%). Of these, 656 (80.4%%) agreed to participate and were randomised, 329 to receive the intervention and 327 to usual care) (Figure 2). Baseline characteristics were similar between the trial groups (Table 1). A total of 107 midwives were trained to deliver the intervention. The first participant was randomised in November 2014 and follow up completed in December 2015.



For peer review only - http://bmjopen.bmj.com/site/about/guidelines.xhtml

**Table 1:** Baseline characteristics by group

| Characteristics | | Randomisation group | |
|---|---|---|---|
| | | Intervention (N=329) | Usual care (N=327) |
| Age (years) | mean (sd) | 29.4 (5.0) | 29.7 (5.2) |
| | Range | 18.3 to 40.6 | 18.0 to 43.0 |
| Height (cm) | mean (sd) | 163.5 (6.5) | 163.3 (6.7) |
| Weight (kg) | mean (sd) | 69.3 (13.8) | 69.7 (13.5) |
| *BMI category* | | | |
| Healthy | | 161 (48.9) | 161 (49.2) |
| Overweight | | 106 (32.2) | 103 (31.5) |
| Obese | | 62 (18.8) | 63 (19.3) |
| BMI (kg/m$^2$) | mean (sd) | 25.9 (4.6) | 26.1 (4.8) |
| IMD quartile | 1 (least deprived) | 34/323 (10.5) | 31/325 (9.5) |
| | 2 | 60/323 (18.6) | 52/325 (16.0) |
| | 3 | 86/323 (26.6) | 96/325 (29.5) |
| | 4 (most deprived) | 143/323 (44.3) | 146/325 (44.9) |
| *Ethnicity* | | | |
| White | | 241/328 (73.5) | 238/327 (72.8) |
| Black Caribbean | | 5/328 (1.5) | 6/327 (1.8) |
| Black African | | 4/328 (1.2) | 5/327 (1.5) |
| Black Other | | 0/328 (0.0) | 1/327 (0.3) |
| Mixed | | 12/328 (3.7) | 7/327 (2.1) |
| Chinese | | 1/328 (0.3) | 3/327 (0.9) |
| Indian | | 10/328 (3.1) | 8/327 (2.5) |
| Pakistani | | 34/328 (10.4) | 39/327 (11.9) |
| Bangladeshi | | 4/328 (1.2) | 4/327 (1.2) |
| Other Asian | | 4/328 (1.2) | 2/327 (0.6) |
| Other | | 13/328 (4.0) | 14/327 (4.3) |
| *Marital status* | | | |
| Married | | 166/315 (52.7) | 195/318 (61.3) |
| Single (living alone) | | 48/315 (15.2) | 33/318 (10.4) |
| Single (living with spouse) | | 95/315 (30.8) | 86/318 (27.0) |
| Widowed | | 0/315 (0.0) | 1/318 (0.3) |
| Divorced/separated (living alone) | | 2/315 (0.6) | 2/318 (0.6) |
| Divorced/separated (living with spouse) | | 2/315 (0.6) | 1/318 (0.3) |
| *Employment status:* | | | |
| In Paid employment | | 218/319 (68.3) | 236/317 (74.5) |
| Student | | 15/319 (4.7) | 5/317 (1.6) |
| Self employed/freelance | | 21/319 (6.6) | 7/317 (2.2) |
| Looking after home/family | | 28/319 (8.8) | 24/317 (7.6) |
| Unemployed | | 35/319 (11.0) | 40/317 (12.6) |
| Sick/disabled | | 1/319 (0.3) | 1/317 (0.3) |
| Retired from paid work | | 0/319 (0.0) | 0/317 (0.0) |
| Other | | 1/319 (0.3) | 4/317 (1.3) |
| Smoking status: | | | |
| Current smoker | | 27/316 (8.5) | 20/317 (6.3) |
| Number of children | mean (sd) n | 0.6 (0.9) 313 | 0.8 (1.1) 315 |
| Attending weight loss programme | | 4/317 (1.3) | 5/317 (1.6) |

Figures are n (%) unless stated otherwise

There was no evidence of a difference in the proportion of women in the intervention and UC groups who gained excessive weight during pregnancy (intervention 27.6% versus usual



For peer review only - http://bmjopen.bmj.com/site/about/guidelines.xhtml

care 28.9%, OR: 0.84, 95% CI: 0.53-0.33, p=0.46). Complete case analysis and different methods of imputation did not alter the results (Table 2). Sub-group analysis (Table 3) showed no evidence that the intervention effect differed by baseline BMI status (p=0.41).

On average women in the intervention group gained 10.3 kg and usual care gained 10.7kg between baseline and 38 weeks of pregnancy. There was no evidence of a difference in the change in weight (kg) during pregnancy (adjusted mean difference -0.42 kg 95% CI: -1.49-0.64) or the amount of weight gained per week of pregnancy between groups (adjusted mean difference -0.01 kg/week 95% CI: -0.038-0.018). There was no evidence of differences in the proportion of women in the groups who gained weight within the IOM guidelines (OR 0.92 95% CI: 0.63-1.32) or less than the minimum the IOM guidance (OR 1.26 95% CI: 0.86-1.83) for gestational weight (Table 3).

Women were doing less physical activity than is recommended for health in pregnancy, and by late pregnancy physical activity had declined, with no difference between groups: mean difference: -4.30 MET hrs/per/week 95% CI:-26.9-18.3 (Table 4).

There was no significant difference between groups in anxiety (mean difference -0.50 95% CI:-1.17-0.16) or depression scores (mean difference -0.59, 95% CI:-1.2-0.05) (Table 3). We planned to assess dietary quality however issues with both data collection and the scoring algorithm meant we were unable to calculate meaningful summary statistics.

No serious adverse events were reported. The numbers of pregnancy complications and adverse neonatal outcomes seemed similar in each group (supplementary Table 1). There was no evidence of intervention contamination in the usual care group. At follow up, seventeen participants in the usual care group responded 'yes' when asked if their midwife talked to them about their weight at their last two appointment. The main reasons were because of concern about weight loss, fluid retention, healthy eating advice, large weight gain and reassurance about weight gain.



For peer review only - http://bmjopen.bmj.com/site/about/guidelines.xhtml

We obtained 214 (65%) of the weight charts from participants' medical notes. Midwives plotted gestational weights and set weight targets in 57% and 50% respectively of scheduled antenatal appointments for the intervention group. Midwives recorded reminding women to weigh themselves weekly at 22% of scheduled appointments. Women in the intervention group weighed themselves on 34% of all weeks. A total of 50.9% (109/214) of women in the intervention group weighed themselves five times or more, 15.9% (34/214) two-four times and 33.2% (71/214) once or less. In the per protocol analyses there was no evidence of a difference between the groups in the proportions who gained excessive gestational weight (supplementary Table 2).



For peer review only - http://bmjopen.bmj.com/site/about/guidelines.xhtml

**Table 2. Comparison of primary outcome (proportion exceeding BMI-related IOM guideline for weight gain during pregnancy) & subgroup analysis.**

| | Intervention | Usual care | | Intervention – Usual care | |
|---|---|---|---|---|---|
| | **n/N (%)** | **n/N (%)** | **Adjusted % difference (95%CI)** | **Adjusted odds ratio (95% CI)** | **P value** |
| **Primary analysis*** Proportion exceeding IOM guideline (multiple imputation of missing 38 week weights) | 81/305 (27.6) | 90/311 (28.9) | -3.5 (-17.8, 10.7) | 0.84 (0.53, 1.33) | 0.46 |
| | | | | | |
| **Sensitivity analysis of proportion exceeding IOM guideline** | | | | | |
| Complete case analysis$ | 51/215 (23.7) | 59/224 (26.3) | -4.8 (-19.8, 10.3) | 0.78 (0.48, 1.26) | 0.31 |
| Imputation with BMI category-specific mean weight# | 85/305 (27.9) | 91/311 (29.3) | -3.1 (-16.0, 9.8) | 0.86 (0.59, 1.26) | 0.44 |
| Imputation with average weight within BMI category related IOM threshold# | 87/305 (28.5) | 93/311 (29.9) | -3.1 (-16.0, 9.9) | 0.86 (0.59, 1.25) | 0.44 |
| | | | | | |
| **Subgroup** | **Intervention** | **Usual Care** | | | |
| **BMI category at recruitment** | **Number exceeding IOM guideline/N (%)+** | **Number exceeding IOM guideline/N (%)+** | **Adjusted odds ratio (95% CI)** | **P value (interaction)** | **-** |
| Healthy weight | 15/148 (10.3) | 22/161 (13.5) | 0.69 (0.22, 2.21 ) | 0.41 | - |
| Overweight | 38/95 (39.8) | 34/93 (36.6) | 1.11 (0.60, 2.04) | | |
| Obese | 31/62 (50.3) | 34/57 (59.6) | 0.69 (0.30, 1.58 ) | | |

Analysis adjusted by Site, BMI category and midwife (random effect). * includes objective and self-reported weights; missing weights imputed using multiple imputation; pooled estimates. $includes objective weights only #Includes objective and self-reported weight.+ includes objective and self-reported weights; missing weights imputed using multiple imputation; pooled estimates



For peer review only - http://bmjopen.bmj.com/site/about/guidelines.xhtml

**Table 3: Comparison of secondary outcome: proportion within or below BMI-related IOM guideline for weight gain during pregnancy.**

| | Intervention | Usual care | Intervention-usual care | | |
|---|---|---|---|---|---|
| | n/N (%) | n/N (%) | Adjusted % difference (95% CI) | Adjusted odds ratio (95% CI) | P value |
| Within IOM guideline$ | 96/305 (31.5) | 108/311 (34.6) | -2.0 (-14.6, 10.6) | 0.92 (0.63, 1.32) | 0.63 |
| Below IOM guideline$ | 125/305 (40.9) | 114/311 (36.5) | 4.9 (-7.4, 17.2) | 1.26 (0.862, 1.827) | 0.24 |

Analysis adjusted by Site, BMI category and midwife (random effect).
$ includes objective and self-reported weights; missing weights imputed using multiple imputation; pooled estimates.



For peer review only - http://bmjopen.bmj.com/site/about/guidelines.xhtml

**Table 4: Comparison of secondary outcomes.**

| | Intervention | | | Usual care | | | Intervention-Usual care | |
|---|---|---|---|---|---|---|---|---|
| | Baseline | 38 weeks | Change | baseline | 38 weeks | Change | | |
| | Mean (sd) n | Mean (sd) n | Mean (sd) n | Mean (sd) n | Mean (sd) n | Mean (sd) n | Adjusted Mean 38 weeks (95% CI)[b] | P value |
| **Weight (kg)[c]** | 71.21 (13.62) 305 | 81.49 (14.35) 305 | 10.28 (5.87) 305 | 71.06 (13.19) 311 | 81.79 (14.35) 311 | 10.73 (6.88) 311 | -0.42 (-1.49, 0.64) | 0.43 |
| **HADS: Anxiety** | 4.88 (3.50) 313 | 5.18 (3.09) 136 | 0.45 (2.82) 136 | 5.15 (3.28) 318 | 5.89 (3.58) 133 | 0.82 (3.33) 132 | -0.58 (-1.25, 0.08) | 0.08 |
| **HADS: Depression** | 3.29 (2.90) 313 | 3.93 (3.04) 136 | 0.75 (2.83) 136 | 3.49 (3.34) 318 | 4.56 (3.04) 133 | 1.29 (3.20) 132 | -0.60 (-1.24, 0.05) | 0.07 |
| **Total physical activity (Met/hrs/wk)** | 283.68 (144.52) 313 | 246.63 (104.97) 136 | -35.02 (115.94) 136 | 278.91 (158.50) 317 | 240.65 (115.26) 132 | -23.43 (117.05) 131 | -4.30 (-26.94, 18.34) | 0.71 |

[b]Adjusted by baseline value, site, BMI category and midwife (random effect). [c] includes objective and self-reported weights; missing weights imputed using multiple imputation; pooled estimates



For peer review only - http://bmjopen.bmj.com/site/about/guidelines.xhtml

## DISCUSSION

There was no evidence that this intervention of weight gain limit setting, regular weighing and feedback delivered by community midwives as part of routine antenatal care was effective. There was however no evidence of psychological harm from the intervention. These findings contribute to the current and on-going debate about whether routine weighing should be re-introduced throughout pregnancy.

Pregnant women have reported that they expect to be weighed during pregnancy and feel it should be part of routine antenatal care.[12, 20] However, three previous trials have investigated the effectiveness of behavioural interventions based on regular weighing to prevent excessive gestational weight gain and none were effective.[7-10] In one, women were advised by a medical student to weigh themselves seven times during pregnancy and were given an IOM weight chart and a table for them to assess their own progress against the targets.[9] In a second, women spent half an hour discussing the importance of healthy weight gain with a research midwife and were encouraged to weigh themselves serially and given an overall weight target, which they were encouraged to discuss with a clinician.[8] However antenatal clinicians were not trained to intervene. In the third women were measured in antenatal clinics and their weight recorded; posters in the clinic were placed to raise women's motivation to stay within the IOM guidelines.[7] The intervention in the present trial was the most complete behavioural intervention to date, comprising both midwife training for routine weighing, setting weight gain limits and feedback, as well as individual advice to women to weigh themselves weekly.

The lack of effectiveness may be attributable to poor intervention delivery. Unlike previous trials, we recorded detailed information about intervention fidelity. In our feasibility trial most midwives commented that they felt the intervention was feasible taking on average about one to two minutes per appointment and it was not perceived as adding substantially to their workload. Midwives also commented that they liked the intervention because it was simple to do and provided them with a legitimate opportunity to raise the topic of gestational weight



For peer review only - http://bmjopen.bmj.com/site/about/guidelines.xhtml

gain. However here, the process evaluation showed only moderate fidelity by midwives in weighing women and setting a target, and little encouragement to women to weigh themselves at home. Only a small proportion of women weighed themselves every week through pregnancy.

Beyond pregnancy, among adults seeking to lose weight, adding regular self-weighing to behavioural weight loss programmes increases effectiveness.[21] The evidence from trials is supported by strong evidence that self-weighing is a key component of the behavioural repertoire of people who are successful at maintaining their weight.[22] However a programme based on self-weighing alone was only minimally effective.[21] We had expected that the greater engagement of women in their own health during pregnancy and concern for the health of their baby might make this a moment when regular weighing would prompt other self-regulatory controls and stimulate effective weight management. In the UK, weighing of women routinely during antenatal care is not recommended and this practice is not part of antenatal care in many other countries, though it is routine in others.[11, 23] NICE noted the lack of evidence of benefit, but also expressed concerns that weighing may cause psychological harm. There was no evidence to indicate any health harms in this trial and other studies suggest that, far from increasing anxiety, it is welcomed by women.[12,20]

Previous research suggested that in many developed countries, the majority of women gain excess gestational weight. Only 28% of the usual care group did so here, not the 60% we assumed would in the sample size calculation. We can only speculate on why the proportion of women gaining excess weight was lower than expected in this trial. It may be due to contamination, with midwives intervening in some unspecified way among women in the usual care group, but we found no evidence of this in feedback from the usual care group and there were no weight charts in the notes of usual care women. Second, perhaps the trial enrolled women who were particularly weight conscious, as 25% of eligible women declined to participate. However, all of those who declined would need to have gained excess weight to reach the frequency of weight gain cited in other studies. One of the attractions of this kind of



For peer review only - http://bmjopen.bmj.com/site/about/guidelines.xhtml

programme is that it is scalable and well-suited to routine care, so that, if effective, it could be applied routinely in prevention in the way that few other interventions can. Future research will need to identify how to engage midwives and women more actively in the process of self-weighing, consider additional behavioural components or identify other interventions and test their effectiveness in this context.

This study has several strengths. Most (~80%) eligible women participated in the trial, meaning the results reflect the impact in the general population. A relatively large proportion of women were recruited from non-White ethnic groups (27%) and/or low socio-economic backgrounds (55%) who are often under-represented in trials. Weight was objectively assessed. Rather than recruit a small number of highly motivated and highly trained midwives, we trained over 100 midwives from a large area of central England to test the intervention in routine practice. To our knowledge this is the first trial where community midwives have delivered an intervention involving setting weight gain limits, regular weighing, encouraging weekly self-weighing, and providing feedback. Unlike trials testing similar interventions, we collected detailed process data on the fidelity of delivery of the intervention and women's adoption of the advice to weigh themselves.

Our findings should also be interpreted in light of some limitations. We estimated that we would follow-up 80% of participants for the primary outcome when calculating the sample size, and achieved 77%. However, only around 42% of women completed the end of pregnancy follow up questionnaires, despite reminders. Although we assessed fidelity, our data on the intervention group were incomplete, with only 65% of weight charts available. This was because some women experienced miscarriage, their notes were not available to the research team, they withdrew from the trial, or removed the charts from their notes. The proportion of women who gained excessive weight was markedly lower than predicted, 30% actual versus 60% predicted from the literature. The sample size was predicated on having 90% power to detect a 15% absolute risk reduction, a relative reduction in incidence of 25%. However, a 25% relative



For peer review only - http://bmjopen.bmj.com/site/about/guidelines.xhtml

reduction from 30% would imply a smaller absolute difference, thus reducing the power of the study below that originally envisaged, which means that a benefit of this treatment programme cannot be confidently ruled out. The development of our intervention may have been enhanced with co-creation with midwives, although the intervention was refined based on the feedback from midwives in our feasibility study.

## CONCLUSION

We did not find evidence to support the value of setting a maximum weight gain limit, regular weighing, and feedback during pregnancy to prevent excessive gestational weight gain. The trial provides reassurance that weighing is not harmful, but in countries where regular weighing is part of usual maternity care, women should be advised that other strategies may be required to prevent excessive gestational weight gain.



For peer review only - http://bmjopen.bmj.com/site/about/guidelines.xhtml

**Figure legends**

Figure 1: Weight chart

Figure 2: Trial flow of participants

For peer review only



For peer review only - http://bmjopen.bmj.com/site/about/guidelines.xhtml

Author affiliations
[1]School of Sport, Exercise and Health Sciences, University of Loughborough, Epinal Way, Loughborough, Leicestershire, LE11 3TU

[2]Institute for Applied Health Research, College of Medical and Dental Sciences, University of Birmingham, Birmingham, B15 2TT

[3]Nuffield Department of Primary Care Health Sciences, University of Oxford, Radcliffe Observatory Quarter, Woodstock Road, Oxford, OX2 6GG

[4]Nuffield Department of Women's and Reproductive Health,  University of Oxford, John Radcliffe Hospital, Headley Way, Oxford, OX3 9DU

[5] Population Health Sciences, Bristol Medical School, , University of Bristol, Canynge Hall, 39 Whatley Road, Bristol, BS8 2PS

For peer review only

Ethics approval and consent to participate
Ethical approval was obtained from NRES Committee West Midlands – South Birmingham: 14/WM/1134, 02/10/14.  All procedures performed in this study were in accordance with the ethical standards of the institutional review board, the American Psychological Association, and with the 1964 Helsinki Declaration. Written informed consent was obtained for all individual participants included in the study.

Patient consent for publication
Not applicable

Availability of the data and material
The datasets used and analysed during the current study are available from the corresponding author on reasonable request, after a period of two years from the date of this publication.

Competing interests
All authors declare that they have no conflict of interest.  All procedures, including the informed consent process, were conducted in accordance with the ethical standards of the responsible committee on human experimentation (institutional and national) and with the Helsinki Declaration of 1975, as revised in 2000.

Funding
The National Institute of Health Research School for Primary Care Research part funded this study.  NIHR CLAHRC-Oxford and NIHR CLAHRC-West Midlands also part funded this study. KJ, CM and SK are part funded by the National Institute for Health Research (NIHR) Collaboration for Leadership in Applied Health Research and Care (CLAHRC) West Midlands. SAJ and PA are NIHR Senior Investigators and supported by the Oxford NIHR Biomedical Research Centre and NIHR CLAHRC Oxford. AR is supported by NIHR Oxford Biomedical Research Centre.  LM works part time for Sensyne Health plc.  The views expressed in this publication are not necessarily those of NHS, the NIHR or the Department of Health. The study was externally peer reviewed for scientific quality.  The funders had no involvement in conducting the research.

Author contributions
AD conceived the original idea for the study with input from PA, KJ, SJ, AL, LM SC, CM and SK.  AD wrote the protocol with contribution from the other authors.  SC was responsible for overseeing data collection.  CO assisted with data collection and provided clinical advice.  AR was responsible for overseeing the writing of the statistical analysis plan.MU conducted the



For peer review only - http://bmjopen.bmj.com/site/about/guidelines.xhtml

analyses. All authors had full access to the data, take responsibility for the integrity of the data and the accuracy of the data analysis, contributed to the interpretation of the results, and reviewed and approved the final manuscript. AD drafted the article and all other authors commented on this draft. AD is the guarantor.

Acknowledgements
We would like to thank the women who took part in this research. We would also like to thank the hospitals and community midwives who agreed to participate in this research; these were from Birmingham Women's NHS Foundation Trust; The Dudley Group NHS Foundation Trust; Oxford University Hospitals NHS Trust and South Warwickshire NHS Foundation Trust. We would also like to thank the PRIME PPI group at Birmingham Women's Hospital for their comments and feedback on the study.

For peer review only

## References

[1] Institute for Medicine. Weight gain in pregnancy: re-examining the guidelines. Washington, DC: The National Academy Press. 2007.

[2] Olson CM, Strawderman MS, Hinton PS, Pearson TA. Gestational weight gain and postpartum behaviors associated with weight change from early pregnancy to 1 y postpartum. *Int J Obes* 2003; **27**: 117–127.

[3] Cohen TR, Koski KG. Limiting excess weight gain in healthy pregnant women: importance of energy intake, physical activity, and adherence to gestational weight gain guidelines. *J Pregnancy* 2013; 787032: 1-6.

[4] Thangaratinam S, Rogozińska E, Jolly K, et al. Effects of interventions in pregnancy on maternal weight and obstetric outcomes: meta-analysis of randomised evidence. *BMJ* 2012; 344: e2088.

[5] Gunderson EP, Abrams B, Selvin S. The relative importance of gestational gain and maternal characteristics associated with the risk of becoming overweight after pregnancy. *Int J obesity Relat Metab Disord* 2000; **24**: 1660-1668.

[6] Scott C, Andersen CT, Valdez N, et al. No global consensus: a cross-sectional survey of maternal weight policies. *BMC Pregnancy Childbirth* 2014; **14**: 1-10.

[7] Brownfoot FC, Davey MA, Kornman L. Routine weighing to reduce excessive antenatal weight gain: a randomised controlled trial. *BJOG* 2016; **123**: 254-261.

[8] McCarthy EA, Walker SP, Ugoni A, Lappas M, Leong O, Shub A. Self-weighing and simple dietary advice for overweight and obese pregnant women to reduce obstetric complications without impact on quality of life: a randomised controlled trial. *BJOG* 2016; **123**: 965-73.

[9] Jeffries K, Shub A, Walker SP, Hiscock R, Permezel M. Reducing excessive weight gain in pregnancy: A randomised controlled trial. *Med J Aus* 2009; **191**: 429-433.

[10] Fealy SM, Taylor RM, Foureur M, et al. Weighing as a stand-alone intervention does not reduce excessive gestational weight gain compared to routine antenatal care: systematic review



For peer review only - http://bmjopen.bmj.com/site/about/guidelines.xhtml

and meta-analysis of randomised controlled trials. *BMC Pregnancy and Childbirth* 2017) **17**:36.

[11] National Institute for Clinical Excellence. Public Health Guidance 18: Weight Management Before, During and After Pregnancy: Public Health Guidance Scope 2010. London: NICE. Accessed at https://www.nice.org.uk/guidance/ph27. Date accessed 7th February 2018.

[12] Daley AJ, Jolly K, Jebb SA, et al. Feasibility and acceptability of regular weighing, setting weight gain limits and providing feedback by community midwives to prevent excess weight gain during pregnancy: randomised controlled trial and qualitative study. *BMC Public Health* 2015; **2**: 35.

[13] Daley AJ, Jolly K, Jebb SA, et al. Effectiveness of regular weighing, weight target setting and feedback by community midwives within routine antenatal care in preventing excessive gestational weight gain: randomised controlled trial. *BMC Obesity (study protocol)* 2016; **3**: 7.

[14] Zigmond AS, Snaith RP. The Hospital Anxiety and Depression Scale. *Acta Psychiatr Scan.* 1983; **67**: 361-70.

[15] Chasan-Taber L, Schmidt MD, Roberts DE, Hosmer D, Markenson G, Freedson PS. Development and validation of a pregnancy physical activity questionnaire. *Med Sci Sport Exer* 2004; **36**: 1750-1760.

[16] Crozier SR, Inskip HM, Barker ME, et al. Development of a 20-item food frequency questionnaire to assess a 'prudent' dietary pattern among young women in Southampton. European Journal of Clinical Nutrition 2010; **64**:99-104.

[17] Kanfer FH. Self-monitoring: methodological limitations & clinical applications. *J Consult Clin Appli* 1970; **35**: 148–158.

[18] The Royal College of Obstetricians and Gynaecologists. Recreational exercise and pregnancy: information for you. 2006. London.

For peer review only



For peer review only - http://bmjopen.bmj.com/site/about/guidelines.xhtml

https://www.rcog.org.uk/globalassets/documents/patients/patient-information-leaflets/pregnancy/recreational-exercise-and-pregnancy.pdf. (Accessed 7th Feb 2018).

[19] Kahan BC, Morris TP. Assessing potential sources of clustering in individually randomised trials. BMC Medical research methodology 2013;13:58.

[20] Allen-Walker V, Mullaney L, Turner MJ, et al. How do women feel about being weighed during pregnancy: A qualitative exploration of the opinions and experiences of postnatal women. *Midwifery* 2017; **49**: 95-101.

[21] Madigan CD, Daley AJ, Lewis AL, Aveyard P, Jolly K. Is self-weighing an effective tool for weight loss: A systematic literature review and meta-analysis. *I J Behav Nutri Phys Activ* 2015; **12**: 104.

[22] Wing RR, Tate DF, Gorin AA, Raynor HA, Fava JL. A self-regulation program for maintenance of weight loss. *N Engl J Med* 2006; **355**: 1563-1571.

[23] Antenatal care: routine care for the healthy pregnant woman. NICE clinical guidelines, No. 62. National Collaborating Centre for Women's and Children's Health (UK). London. RCOG Press; 2008. https://www.nice.org.uk/guidance/cg62/evidence/evidence-tables-from-the-2003-version-pdf-196748322. Date accessed 23rd June 2017.

For peer review only



For peer review only - http://bmjopen.bmj.com/site/about/guidelines.xhtml

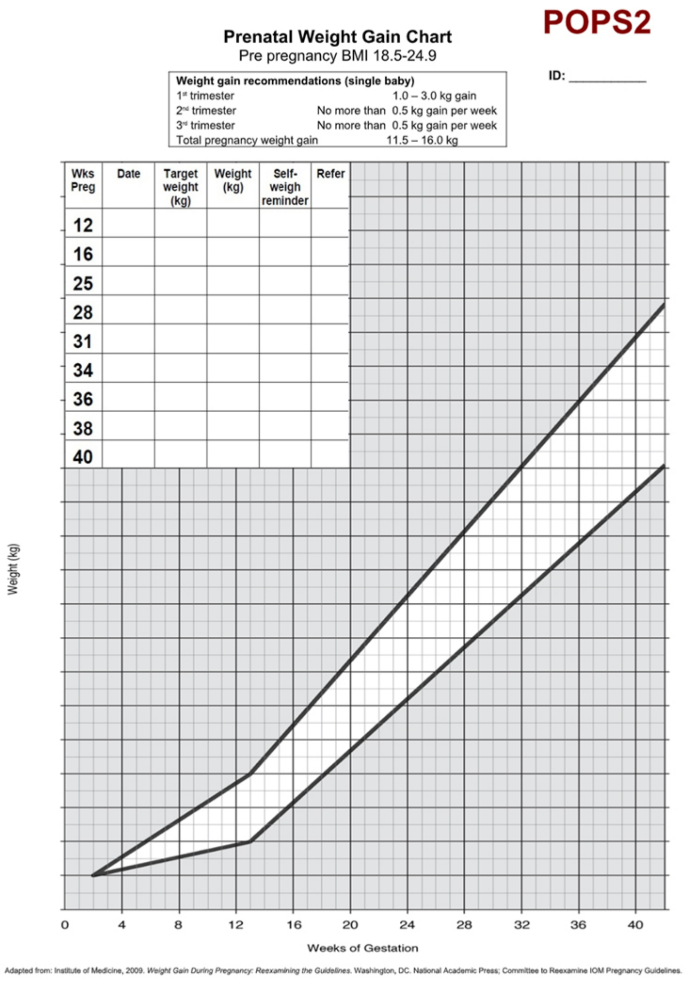


Figure 1 Weight chart

90x90mm (300 x 300 DPI)

**Figure 2: Trial flow**

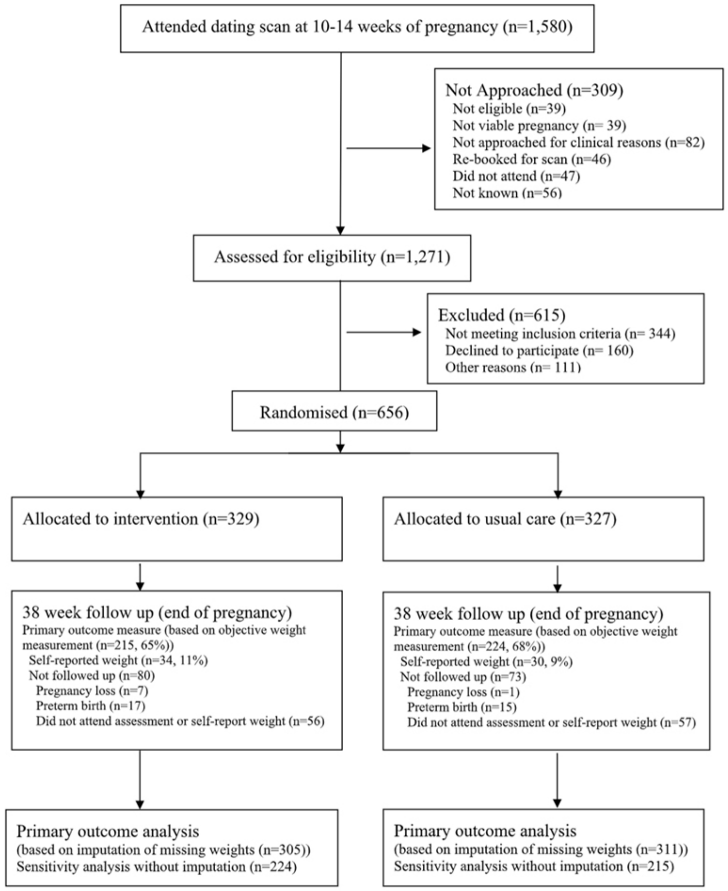


Trial flow

90x90mm (300 x 300 DPI)

**Supplementary Table 1: Maternal and neonatal complications by group**

| | | Intervention | Usual care |
|---|---|---|---|
| **Mother:** | | N (%) | N (%) |
| Caesarean section | | 68/304 (22.4) | 69/302 (22.8) |
| Length of inpatient stay | mean (sd)<br>median (IQR) | 1.7 (1.5) 305<br>1.0 (1.0 to 2.0) | 1.7 (1.7) 306<br>1.0 (1.0 to 2.0) |
| Maternal ICU admission | | n/a | n/a |
| Preeclampsia | | 6/315 (1.9) | 8/317 (2.5) |
| Pregnancy induced hypertension | | n/a | n/a |
| Gestational diabetes | | 12/315 (3.8) | 17/317 (5.4) |
| Maternal sepsis | | n/a | n/a |
| Preterm delivery | | 17/304 (5.6) | 14/302 (4.6) |
| Miscarriage | | 5/317 (1.6) | 1/316 (0.3) |
| Stillbirth | | 0/316 (0) | 1/316 (0.3) |
| Shoulder dystocia | | 3/312 (1.0) | 2/314 (0.6) |
| **Baby:** | | | |
| Treatment for jaundice | | 34/312 (10.9) | 27/314 (8.6) |
| Low Apgar score (<7) at 1 min<br>Low apgar score (<7) at 5 mins | | n/a<br>4/254 (1.6) | n/a<br>1/249 (0.4) |
| Admission to NICU | | 26/261 (10.0) | 21/262 (8.0) |
| Neonatal death | | n/a | n/a |
| Neonatal sepsis | | n/a | n/a |
| Gestational age (wks) | mean (sd)<br>median (IQR) | 39.2 (2.1) 304<br>40 (39-40) | 39.3 (1.6) 302<br>40 (38-40) |
| Birth weight (g) | mean (sd)<br>median (IQR) | 3348.6 (567.1) 304<br>3373.5 (3060-3665) | 3401.8 (550.7) 301<br>3460 (3040-3745) |

For peer review only

Supplementary Table 2: Per-protocol analysis

| | Intervention | Usual care | Intervention-Usual care | | |
|---|---|---|---|---|---|
| | **Number exceeding IOM guideline/N$(%)** | **Number exceeding IOM guideline/N$(%)** | **% (95% CI)*** | **Adjusted odds ratio (95% CI)*** | **P value** |
| Women recorded weight weekly >70% of time during pregnancy | 7/46 (15.2) | 67/254 (26.4) | -9.2 (-26.3, 8.0) | 0.58 (0.23, 1.47) | 0.25 |
| Midwife overall accuracy for >70% appointments | 8/42 (19.0) | 67/254 (26.4) | -5.9 (-28.0, 16.2) | 0.71 (0.27, 1.88) | 0.49 |
| Women weighing themselves 5 or more times during pregnancy | 18/95 (18.9) | 67/254 (26.4) | -7.0 (-23.2, 9.2) | 0.68 (0.35, 1.33) | 0.26 |

$ Includes objective and self-reported weights
*adjusted by Site, BMI category and midwife (random effect)

For peer review only

# CONSORT 2010 checklist of information to include when reporting a randomised trial*

| Section/Topic | Item No | Checklist item | Reported on page No |
|---|---|---|---|
| **Title and abstract** | | | |
| | 1a | Identification as a randomised trial in the title | Title page |
| | 1b | Structured summary of trial design, methods, results, and conclusions (for specific guidance see CONSORT for abstracts) | 1 |
| **Introduction** | | | |
| Background and objectives | 2a | Scientific background and explanation of rationale | 3-4 |
| | 2b | Specific objectives or hypotheses | 4 |
| **Methods** | | | |
| Trial design | 3a | Description of trial design (such as parallel, factorial) including allocation ratio | 5 |
| | 3b | Important changes to methods after trial commencement (such as eligibility criteria), with reasons | N/A |
| Participants | 4a | Eligibility criteria for participants | 5-6 |
| | 4b | Settings and locations where the data were collected | 5 |
| Interventions | 5 | The interventions for each group with sufficient details to allow replication, including how and when they were actually administered | 7-8 |
| Outcomes | 6a | Completely defined pre-specified primary and secondary outcome measures, including how and when they were assessed | 6-7 |
| | 6b | Any changes to trial outcomes after the trial commenced, with reasons | N/A |
| Sample size | 7a | How sample size was determined | 9 |
| | 7b | When applicable, explanation of any interim analyses and stopping guidelines | N.A |
| Randomisation: | | | |
| Sequence generation | 8a | Method used to generate the random allocation sequence | 6 |
| | 8b | Type of randomisation; details of any restriction (such as blocking and block size) | 6 |
| Allocation concealment mechanism | 9 | Mechanism used to implement the random allocation sequence (such as sequentially numbered containers), describing any steps taken to conceal the sequence until interventions were assigned | 6 |
| Implementation | 10 | Who generated the random allocation sequence, who enrolled participants, and who assigned participants to interventions | 6 |
| Blinding | 11a | If done, who was blinded after assignment to interventions (for example, participants, care providers, those | N/A |

For peer review only

| | | | |
|---|---|---|---|
| | | assessing outcomes) and how | |
| | 11b | If relevant, description of the similarity of interventions | N.A |
| Statistical methods | 12a | Statistical methods used to compare groups for primary and secondary outcomes | 9-10 |
| | 12b | Methods for additional analyses, such as subgroup analyses and adjusted analyses | 9-10 |
| **Results** | | | |
| Participant flow (a diagram is strongly recommended) | 13a | For each group, the numbers of participants who were randomly assigned, received intended treatment, and were analysed for the primary outcome | 11 |
| | 13b | For each group, losses and exclusions after randomisation, together with reasons | 11 |
| Recruitment | 14a | Dates defining the periods of recruitment and follow-up | 11 |
| | 14b | Why the trial ended or was stopped | N/A |
| Baseline data | 15 | A table showing baseline demographic and clinical characteristics for each group | 19 |
| Numbers analysed | 16 | For each group, number of participants (denominator) included in each analysis and whether the analysis was by original assigned groups | 12-13 |
| Outcomes and estimation | 17a | For each primary and secondary outcome, results for each group, and the estimated effect size and its precision (such as 95% confidence interval) | 12-13 |
| | 17b | For binary outcomes, presentation of both absolute and relative effect sizes is recommended | 12-13 |
| Ancillary analyses | 18 | Results of any other analyses performed, including subgroup analyses and adjusted analyses, distinguishing pre-specified from exploratory | 12-13 |
| Harms | 19 | All important harms or unintended effects in each group (for specific guidance see CONSORT for harms) | 13 |
| **Discussion** | | | |
| Limitations | 20 | Trial limitations, addressing sources of potential bias, imprecision, and, if relevant, multiplicity of analyses | 12 |
| Generalisability | 21 | Generalisability (external validity, applicability) of the trial findings | 10-12 |
| Interpretation | 22 | Interpretation consistent with results, balancing benefits and harms, and considering other relevant evidence | 10-12 |
| **Other information** | | | |
| Registration | 23 | Registration number and name of trial registry | 1 |
| Protocol | 24 | Where the full trial protocol can be accessed, if available | 5 |
| Funding | 25 | Sources of funding and other support (such as supply of drugs), role of funders | 15 |

*We strongly recommend reading this statement in conjunction with the CONSORT 2010 Explanation and Elaboration for important clarifications on all the items. If relevant, we also recommend reading CONSORT extensions for cluster randomised trials, non-inferiority and equivalence trials, non-pharmacological treatments, herbal interventions, and pragmatic trials. Additional extensions are forthcoming: for those and for up to date references relevant to this checklist, see www.consort-statement.org.


For peer review only - http://bmjopen.bmj.com/site/about/guidelines.xhtml
For peer review only
